# Analysis of tarantula skeletal muscle protein sequences and identification of transcriptional isoforms

**DOI:** 10.1186/1471-2164-10-117

**Published:** 2009-03-19

**Authors:** Jingui Zhu, Yongqiao Sun, Fa-Qing Zhao, Jun Yu, Roger Craig, Songnian Hu

**Affiliations:** 1 Key laboratory of Genome Sciences and Information, Beijing Institute of Genomics, Chinese Academy of Sciences, Beijing, PR China; 2 University of Massachusetts Medical School, Worcester, MA, USA; 3 The Graduate University of Chinese Academy of Sciences, Beijing, PR China

## Abstract

**Background:**

Tarantula has been used as a model system for studying skeletal muscle structure and function, yet data on the genes expressed in tarantula muscle are lacking.

**Results:**

We constructed a cDNA library from *Aphonopelma sp*. (Tarantula) skeletal muscle and got 2507 high-quality 5'ESTs (expressed sequence tags) from randomly picked clones. EST analysis showed 305 unigenes, among which 81 had more than 2 ESTs. Twenty abundant unigenes had matches to skeletal muscle-related genes including actin, myosin, tropomyosin, troponin-I, T and C, paramyosin, muscle LIM protein, muscle protein 20, a-actinin and tandem Ig/Fn motifs (found in giant sarcomere-related proteins). Matches to myosin light chain kinase and calponin were also identified. These results support the existence of both actin-linked and myosin-linked regulation in tarantula skeletal muscle.

We have predicted full-length as well as partial cDNA sequences both experimentally and computationally for myosin heavy and light chains, actin, tropomyosin, and troponin-I, T and C, and have deduced the putative peptides. A preliminary analysis of the structural and functional properties was also carried out. Sequence similarities suggested multiple isoforms of most myofibrillar proteins, supporting the generality of multiple isoforms known from previous muscle sequence studies. This may be related to a mix of muscle fiber types.

**Conclusion:**

The present study serves as a basis for defining the transcriptome of tarantula skeletal muscle, for future *in vitro *expression of tarantula proteins, and for interpreting structural and functional observations in this model species.

## Background

Skeletal muscle is the key tissue used by animals for locomotion and other movements of the body. It thus has important implications in physiology and medicine. The contractile machinery consists of thick filaments, composed of myosin and myosin-binding proteins, thin filaments, composed of actin and the thin-filament associated proteins troponin and tropomyosin, Z-line proteins, and giant proteins involved in assembly of the filaments into the contractile units called sarcomeres [1]. Within a single adult skeletal muscle, distinct muscle fiber types, with different sets of protein isoforms and different functional properties, can be found side by side [2]. This heterogeneity enables a flexible contractile response. From studies of *Drosophila, C. elegans*, bivalvia, decapod crustaceans, and other invertebrates, it is recognized that invertebrate muscle genes and proteins show numerous variations on the common theme of thick and thin filament assembly and interaction [3]. These variations have played an important role in revealing common themes and how specialization allows animals to adapt to particular needs.

Tarantula skeletal muscle has emerged as a key model system for understanding the structural characteristics of muscle thick filaments. The thick filaments have a highly ordered array of myosin heads, which is easier to study structurally than that in the more complex and less stable filaments found in vertebrate skeletal muscle. Studies of tarantula thick filaments have led to a new understanding of thick filament assembly and regulation [4]. These newly gained insights have been shown to be directly relevant to myosin function in vertebrate muscles. In addition, tarantula, like most invertebrates, is dually-regulated, via both actin-linked and myosin-linked systems [5,6], and its thin filaments have provided insights into actin-myosin interaction and regulation [7]. Despite these structural advances, sequence information on tarantula muscle proteins, which could provide a critical complement to structural and functional knowledge of this muscle, has been lacking: no tarantula muscle-related sequences, either mRNA or protein, are yet in public databases. Furthermore, the subphylum chelicerata to which the tarantula belongs has been infrequently sampled for muscle research, and there are only a few records of muscle proteins.

Expressed sequence tags (ESTs) are short single-pass sequence reads generated from either 5' or 3' end of cDNAs. They provide a quick and inexpensive route for discovering new genes and obtaining data on gene expression [8]. To obtain a rough picture of the genes expressed in tarantula skeletal muscle, we constructed a non-normalized cDNA library from *Aphonopelma *sp. leg muscle, and randomly generated and analyzed 2507 5'ESTs. We have identified transcripts of skeletal muscle-related genes, most with more than one isoform, and predicted full-length as well as partial cDNA sequences of major myofibrillar proteins based on bioinformatics analysis, pair-wise end sequencing, and primer walking. We also provide a preliminary analysis of the properties of the peptides deduced from the above transcripts.

We believe this to be the first report of an EST strategy applied to tarantula skeletal muscle. The sequence data should greatly facilitate further structural and functional research.

## Results and discussion

### Overview of ESTs from the *Aphonopelma *skeletal muscle cDNA library

**Figure 1:**
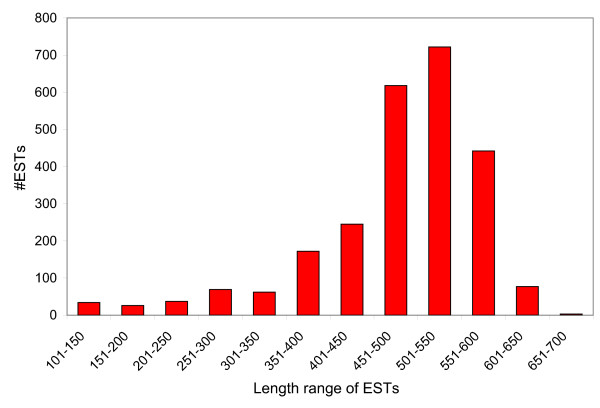
**Read length distribution of 5'ESTs from Aphonopelma skeletal muscle**. A total of 2507 high quality 5'ESTs were analyzed in the current study. Abscissa is the sequence length in 50 bp intervals, while the ordinate is the number of ESTs.

From the tarantula skeletal muscle cDNA library that we constructed, we sequenced over 2609 randomly picked clones from the 5'end. After removal of vector and poor quality sequences, 2507 high-quality ESTs remained, averaging 478 bp (Figure 1). The ESTs obtained in this work have been deposited in dbEST [**GenBank: **FC823253–FC825759] (Additional File 1).

The high-quality ESTs were first assembled into contigs and singletons (see Methods for definitions), which were subsequently searched against GenBank nonredundant protein (Nr) database. After the grouping of contigs and singletons with the same best Nr hit, 305 unigenes (see Methods for definition) were identified in total, among which 81 had more than 2 ESTs. 2014 5'ESTs had significant matches to Nr at 1e-5 cutoff, accounting for 80.33% of the total sequences (Additional File 2). Based on searches against Uniprot database, the same 2014 5' ESTs were annotated. Among the 493 5'ESTs without Nr or Uniprot hits, 40 had significant matches to the nonredundant nucleotide (Nt) database at 1e-10. 69 of the 5'ESTs without either Nr or Nt hits had significant matches to chelicerate EST at 1e-10, with a great majority (67 out of 69) matching to ESTs from *Acanthoscurria gomesiana *(another tarantula) hemocyte normal and normalized libraries [9], suggesting that these ESTs encode novel transcripts expressed in tarantula. Since the *Aphonopelma *muscle cDNA library was neither subtracted nor normalized, the unigene size or EST counts would be expected to reflect the relative abundance of the corresponding mRNA populations.

**Figure 2:**
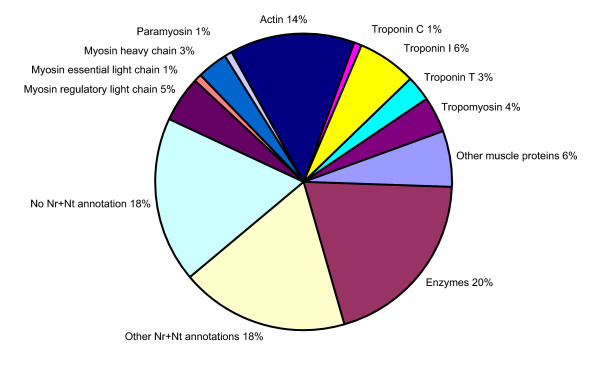
**Functional annotation of the high quality 5'ESTs. **The relative proportion of different types of transcripts: myosin (further divided into regulatory light chain, essential light chain and heavy chain), paramyosin, actin, troponin (further divided into troponin C, I and T), tropomyosin, other muscle proteins, enzymes, other Nr and Nt annotation, no Nr and Nt annotation. Nr and Nt are the nonredundant protein and nucleotide databases in GenBank respectively.

The functional annotation of all ESTs is depicted in Figure 2. The most abundant transcripts were arginine kinase homologues involved in maintaining high cytosolic levels of ATP. ESTs coding for the major myofibrillar proteins, including actin, myosin heavy chain and light chains, troponin I, C and T, and tropomyosin, were well represented, accounting in total for 37% of the 5'ESTs sequenced. Each of these proteins was present in more than one variant. This indicates the existence of different isoforms of these proteins in tarantula, as detailed later. Other proteins related to the contractile machinery and its assembly were also represented, including paramyosin, titin-like proteins, LIM proteins, actin-binding proteins, myosin light chain kinase (MLCK) and calmodulin, and sarcoplasmic reticulum proteins. Other common nonmyofibrillar proteins included enzymes involved in energy metabolism, transposase, hemocyanin and structural constituents of ribosomes (Additional File 2).

Most invertebrate thick filaments contain a core of paramyosin [10-12], as well as small versions of titin-like proteins consisting of immunoglobulin-like (Ig) and fibronectin-like (Fn) domains, which contribute to the mechanical, structural and developmental properties of these muscles[13]. Consistent with these generalizations, several of the tarantula ESTs showed significant homology to paramyosin[12], and matches to repeated, sequential Ig and Fn motifs characteristic of titin-like proteins were also found by InterProScan. However, it was difficult to determine which specific protein(s) these Ig/Fn ESTs encoded without additional evidence from non-Ig/Fn domains. BLASTX searches against Swiss-Prot showed 7 clones with significant matches to human titin and 6 to *Drosophila melanogaster *titin, though the identities were low (30–57%) (Additional File 2). This supports the conclusion that there are titin-like proteins in tarantula skeletal muscle.

Transcripts homologous to LIM proteins were also abundant. The LIM domain is a zinc-binding motif found in a growing number of eukaryotic proteins, which regulates gene expression, cytoarchitecture, cell adhesion, cell motility and signal transduction [14]. Several LIM proteins are found in muscle Z-lines [15,16], which function to link together actin filaments from adjacent sarcomeres. 72 EST clones showed significant homology to muscle LIM protein1, which plays a role in myogenesis and is associated with the muscle sarcomere in *Drosophila melanogaster *[17]. There were also 26 clones with significant hits to other LIM proteins, including four and a half LIM domain proteins, PDZ and other LIM domain-containing proteins (Additional File 2). The LIM/PDZ proteins may associate with the actin cytoskeleton or myofibrillar Z-lines and/or influence the actin cytoskeleton in tarantula skeletal muscle as reported in other species [18].

Calponin-homology domains that belong to a superfamily of actin-binding domains [19] are found in a number of muscle proteins, and were also significant in the tarantula muscle transcriptome. The tarantula muscle ESTs were enriched in transcripts homologous to muscle protein 20 (mp20) which belongs to the calponin family and has 2 regions with calcium-binding sites, with 55 clones in total. mp20 is a muscle-specific protein expressed in all the muscles of *Drosophila melanogaster*, both larval and adult, with the exception of the flight muscles [20]. There were also 2 singletons with homology to calponin-like actin binding domains and SM22/calponin domains (Additional File 2). Transcripts with significant matches to the major Z-line protein alpha-actinin, which cross-links actin filaments and titin molecules in the Z-disc [21], were also present in tarantula muscle (Additional File 2). Alpha-actinin has both calponin-homology domains and spectrin-like domains.

Matches to myosin light chain kinase (MLCK) and calmodulin as well as the sarcoplasmic reticulum (SR) Ca^2+^-ATPase were identified (Additional File 2). The sarcoplasmic reticulum Ca^2+^-ATPase is a pump that transports calcium ions from the cytoplasm into the SR, bringing about relaxation of a contracting muscle [22]. Myosin light chain kinase regulates contraction in many muscles by phosphorylating the myosin regulatory light chain, a process that is calcium-calmodulin dependent. Thus, our preliminary analysis confirms the existence of myosin-linked (in addition to actin-linked, supported by the troponin-tropomyosin complex) regulation in tarantula skeletal muscle [5], as seen in most invertebrate skeletal muscles.

### Identification and analysis of transcriptional isoforms of the major myofibrillar proteins

#### Myosin structure

Muscle myosin (myosin II) is a hexamer that contains two heavy chains (MHCs) and two pairs of light chains [1]. The C-terminal halves of the two MHCs form an α-helical coiled-coil tail that, with other tails, forms the backbone of the assembled thick filament. The N-terminal halves separate into two heads, each consisting of a globular motor domain, with ATPase and actin-binding activity, and an α-helical neck region (or light chain domain), which binds one regulatory light chain (RLC) and one essential light chain (ELC). The cyclic interaction of the myosin heads with actin in the thin filaments draws the filaments past one another to generate muscle contraction. The light chain domain acts as a lever that converts small movements in the converter region of the motor domain into the larger movements of the lever that are responsible for pulling on actin [23]. In addition to its structural role in the lever, the RLC can be phosphorylated in most species (as mentioned above), thereby regulating or modulating contraction in response to changes in intracellular Ca^2+ ^levels [1].

#### Myosin Regulatory light chain

Two contigs encoding RLC homologues were identified. One was highly abundant, consisting of ESTs from 125 clones and containing the complete open-reading frame (ORF) for 196 amino acids (aa). The other consisted of ESTs from 5 clones, and contained the complete open-reading frame for 235 aa. The two putative full-length RLCs showed 56% identity. We conclude that there are at least two RLC isoforms in tarantula skeletal muscle, and that the full-length peptide deduced from the contig of 125 clones is the major expressed isoform (designated as MLR1_As, and referred to below as the major RLC), while the other (MLR2_As) is the minor isoform (Figure S1 and S2, Additional File 3).

The major RLC mRNA would lead to a polypeptide of 21.8 kDa, with a theoretical pI of 4.74. The minor RLC was 39 aa longer than the major RLC. It would produce a polypeptide of about 26 kDa, and a theoretical pI of 4.66. Comparative sequence analysis revealed that both RLC isoforms are most similar to myosin II regulatory light chain from *Avicularia avicularia *(pinktoe tarantula), with 95% identity for the major RLC and 56% identity for the minor RLC.

**Figure 3:**
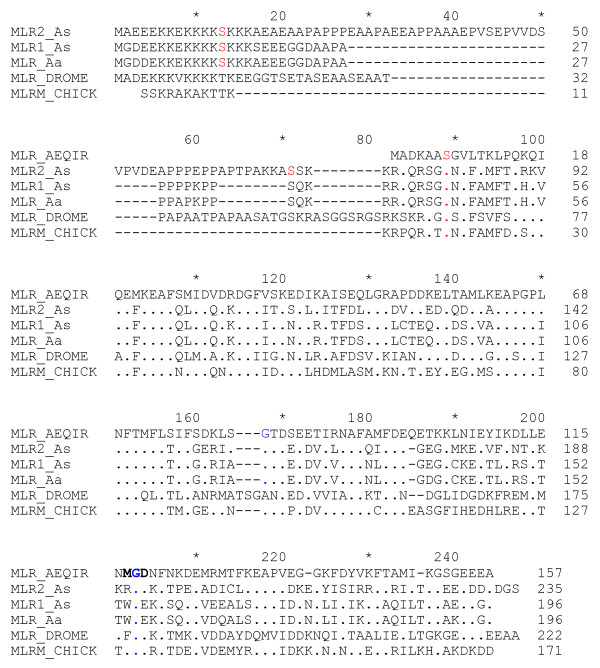
**Alignment of the tarantula RLC isoforms to scallop, *Drosophila *and chicken smooth muscle RLCs**. The abbreviations and accession numbers for the RLC sequences aligned are: MLR_AEQIR, *Aequipecten irradians *(Bay scallop) striated adductor muscle RLC (P13543); MLR1_As, *Aphonopelma sp*. (Tarantula) skeletal muscle RLC major isoform; MLR2_As, *Aphonopelma sp*. (Tarantula) skeletal muscle RLC minor isoform; MLR_Aa, *Avicularia avicularia *(pinktoe tarantula) myosin II RLC (ABW76151); MLR_DROME, *Drosophila melanogaster *(Fruit fly) myosin RLC 2 (P18432); MLRM_CHICK, *Gallus gallus *(Chicken) myosin RLC2 smooth muscle major isoform (P02612). Dot indicates identity with the top sequence; Dash indicates a gap inserted for spacing purposes. The putative MLCK phosphorylation sites of tarantula RLCs and the conserved MLCK phosphorylation sites across the species are labeled in red. The Glycine residues important in the interaction of the RLC with the MHC in scallop and conserved in tarantula and chicken are labeled in blue. The MGD motif involved in binding both MHC and ELC in scallop RLC is in bold. The conserved Glycine residues in the MGD motif across the species are in blue.

Many invertebrate species show N-terminal extensions on their RLCs when compared with vertebrate skeletal and smooth muscle RLCs (for example, *Drosophila melanogaster *has a 46 aa long extension). Both tarantula RLCs showed such extensions (Figure 3). These RLC extensions are similar to ELC N-terminal extensions in vertebrates, which bind actin. In both vertebrates and invertebrates, these N-terminal extensions are thought to play a similar role, maintaining a link between the thick and thin filaments in parallel with the myosin cross-bridge [24,25]. Our results are consistent with a similar role in tarantula muscle.

In earlier work, it was concluded that the tarantula RLC occurred as two major species with the same molecular weight (26 kDa) but different charges [5]. Further study suggested that there was in fact only one major RLC, which could be phosphorylated at two sites [26]. Our EST analysis supports the view that two RLC species are expressed in tarantula muscle and, in addition, both can be phosphorylated at more than one site. The low expression of the minor form may have precluded its detection by SDS-PAGE. The predicted 21.8 kDa molecular weight of the major RLC isoform is 4 kDa less than 26 kDa reported based on SDS-PAGE [5], suggesting anomalous migration of the RLC on electrophoresis [27].

Two putative sites for phosphorylation of the major tarantula RLC by MLCK were predicted [28], consistent with earlier conclusions [26]. These were at Ser13 and Ser45. In the minor RLC, three putative MLCK phosphorylation sites were identified: Ser13, Ser 71 and Ser81. Ser45 of the major RLC and Ser81 of the minor RLC are located in the sequence RXXSXBB ("S" is the phophorylatable Serine, "X" is any amino acid and "B" is any hydrophobic amino acid A, V, F, I, or L) important for substrate recognition by smooth muscle MLCKs and conserved across most species [29], so it is likely that they play a role in phosphorylation-based regulation of myosin activity (Figure 3). How closely the other predicted phosphorylation sites in tarantula correspond to those occurring *in vivo *requires experimental investigation. The tarantula RLC results are similar to those of *Limulus*, a close evolutionary relative of tarantula, which has two skeletal muscle RLCs with different molecular weights, both of which can be phosphorylated at one or two sites [30-32].

In molluscan myosins regulated by Ca^2+ ^binding to the ELC, a Gly in the middle of the RLC has been shown to be important in the interaction with the heavy chain. There is also an MGD motif that is important in the interaction between the RLC, ELC and HC, stabilizing the calcium-binding motif on the ELC [33,34]. The G in the MGD motif is found in RLCs of myosins regulated by both RLC phosphorylation and Ca^2+ ^binding. Sequence alignment shows these two Gly sites to be conserved in the tarantula RLCs, while the other two residues (WGE and RGD) at the positions homologous to the MGD differ from the common patterns seen in other muscles [35] (Figure 3).

There is one functional cation-binding helix-loop-helix motif (EF-domain) in some RLCs [35]. Residues at positions 1, 3, 5, 7, 9 and 12 of the loop are responsible for binding the cation, and are conventionally designated X, Y, Z, -Y, -X and -Z. Of the sites responsible for putative cation binding in the two tarantula RLCs, residues at X, Y, Z and -Z are all D, which could chelate Ca^2+^, while -Y is F and -X is S, suggesting that tarantula RLCs do not bind cation on the EF-domain [35].

#### Myosin Essential light chain

Two ELC isoforms are expressed in vertebrate fast skeletal muscle, differing by approximately 40 amino acids at their amino-terminal. Multiple ELC isoforms have also been found in some invertebrates such as scallop and *Drosophila*[3], although not previously in tarantula.

We found three contigs homologous to ELCs. One consisted of 12 clones and contained a complete open-reading frame (MLE1_As). Another contig of 12 clones encoded the C-terminal 81 aa of a second putative ELC (MLE2_As), and the other, of 2 clones, encoded the N-terminal 87 aa of another putative ELC. The two putative ELC fragments showed only 62% and 51% identity to MLE1_As respectively. Thus there appear to be at least 3 ELC isoforms in tarantula skeletal muscle.

The MLE1_As isoform is 156 amino acids in length, which would lead to a polypeptide of 17.6 kDa, with a theoretical pI of 4.62. As with other invertebrates, this ELC lacked the Pro-Ala rich N-terminal extension of the long vertebrate ELC isoform, and was more similar to the short isoform. Comparative sequence analysis revealed that the ELC was most similar to the alkali light-chain of tick (*Haemaphysalis qinghaiensis*) myosin, with 71% identity.

Analysis of ESTs from hemocytes of another tarantula, *Acanthoscurria gomesiana *showed two ELC homologues, one of which is 95% identical (7 substitutions, including one stop codon most probably due to sequencing error) to the MLE1_As and likely to be a skeletal muscle isoform too (Figure S1, Additional File 4), while the other represented by more ESTs deviated from MLE1_As and is likely to be a non-muscle isoform. Thus it appears that the skeletal muscle isoform of the ELC is expressed in nonmuscle tissues too.

ELCs belong to the EF-hand family of calcium binding proteins. However, as with most other striated muscle ELCs, the tarantula ELC lacked the calcium-binding residues conserved in scallop[36] (Figure S1, Additional File 4).

#### Myosin Heavy chain

The myosin II heavy chain generally has a MW of about 220 kDa and more than 1900 aa. It can be divided into S1 (or subfragment1, the head), S2 (subfragment 2, the proximal one third of the tail), and LMM (light meromyosin, the distal two thirds of the tail).

**Figure 4:**
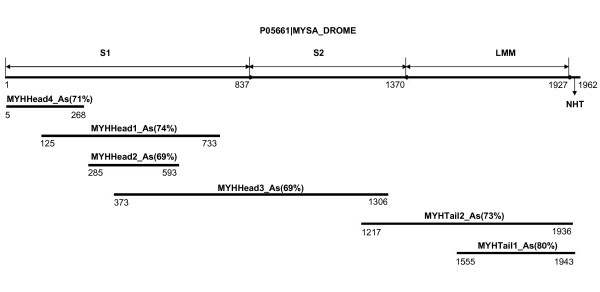
**Schematic of the tarantula myosin II heavy chain fragments**. The tarantula myosin II heavy chain fragments obtained from EST assembly are placed immediately below the relevant portions of the linear map of *Drosophila *muscle myosin II heavy chain. The numbers under each myosin II heavy chain fragment indicate the locations of the start and end positions of its best match in *Drosophila *muscle myosin II heavy chain. The identity between each myosin II heavy chain fragment and *Drosophila *muscle myosin II heavy chain is shown in parentheses. Abbreviations: P05661|MYSA_DROME, *Drosophila *muscle myosin II heavy chain; S1, myosin subfragment 1; S2, myosin subfragment 2; LMM, L-meromyosin; NHT, non-helical tailpiece.

We found 85 5'ESTs homologous to different parts of the muscle isoform of *Drosophila melanogaster *myosin heavy chain. There were 10 contigs and 1 singleton homologous to the myosin heavy chain. In all, there were at least 4 variants of the myosin heavy chain head and 2 major variants of the tail in tarantula skeletal muscle. So far no complete S1, S2 or LMM has been obtained, although sequences encompassing several regions of these domains were determined (Figure 4). The MHC fragments containing portions of the head in our study appeared to belong to class II type myosin, since they showed conservation of sequences specifically found in the myosin II motor domain [37]. All the fragments that included part of the tail showed coiled-coil domains and one that reached the C-terminus contained a non-helical tailpiece (both characteristics of myosin II) rich in prolines. The non-helical tailpiece has been suggested to play an essential role in the initiation and modulation of filament assembly [38]. A myosin N-terminal SH3-like domain was found in one contig (MYHHead4_As). The fragments did not show any domains characteristic of other MHC classes [37].

The most abundant contig was composed of 115 5', 3' and primer walking ESTs. It matches the C-terminal 389 aa of the muscle isoform of *Drosophila melanogaster *MHC and possesses the stop codon and 3'UTR. It is designated as MYHTail1_As. Another contig composed of 11 ESTs and designated as MYHTail2_As encodes the C-terminal 719 aa region. However, it lacks the stop codon. Both contigs showed highest homology to the muscle isoform of *Drosophila melanogaster *myosin heavy chain, with 80% identity for MYHTail1_As and 73% for MYHTail2_As. The deduced peptide sequences of the two tail isoforms are 86% identical and 93% similar. As with all myosin II tails, both sequences showed a high predicted propensity to form alpha-helical coiled coils [38]. The typical 28 aa repeats in myosin II tails have been reported to be interrupted by the insertion of extra residues called "skip residues" at 3 positions in smooth and non-muscle myosin II and 4 positions in striated muscle myosin II [39,40]. These skip residues cause local distortions in the pitch or stability of the coiled coil, which may have important effects on the packing of myosin tails in the filament backbone [40,41]. Based on McLachlan and Karn's [39,40]designation, all four skip residues in striated muscle myosin were also found in the tarantula sequences: the last three (Glu, Glu and Gly) in both tarantula tail sequences, and the first in the MYHHead3_As contig (which also contains the first region of the tail; see below; Figure S3, Additional File 4).

We found five contigs with homology to different regions of the S1 head of myosin II. One contig matches the 5–268 aa region of *Drosophila *muscle MHC, contains the start codon and 5'UTR, and is designated as MYHHead4_As. Another contig, 2815 bp long and designated MYHHead3_As, matches the 373–1306 aa region of the *Drosophila *heavy chain. We designed primers to explore whether it was possible to bridge the two contigs by PCR. A PCR product of about 2 kb was obtained, which matched the 125–733 aa region of the *Drosophila *heavy chain, yet differed from the above two contigs, and contained a region almost the same as another contig composed of 8 ESTs previously identified instead. The PCR product encoded the longest partial S1, and was designated MYHHead1_As. Another contig, matching the 285–593 aa region of *Drosophila *was designated MYHHead2_As. These head sequences shared 78%–86% identity with each other, 69–74% identity with myosin heavy chain of *Drosophila melanogaster *and around 60% identity with scallop.

Analysis of the partial myosin head sequences in tarantula showed that the conserved residues were more concentrated in the ATP-binding region than the actin-binding regions, and loop regions were the least conserved. Two sequence isoforms of loop I region and 1 sequence isoform of loop II region were identified from our partial sequences. There are a few prolines in one loop I variant and several Gly in loop II. Three Trp residues in the ELC binding region of MYHHead3_As substituted for nonaromatic aa in scallop myosins, while 2 Trp residues in the RLC binding region in scallop myosins were substituted by two other aromatic aa in MYHHead3_As. The sequences between SH2 and SH1 are conserved completely in all tarantula isoforms (Figure S2, Additional File 4).

#### Paramyosin

Paramyosin is an α-helical coiled coil molecule, comparable to a shortened myosin tail, found in the core of thick filaments of invertebrate muscles, both striated and smooth. The myosin/paramyosin ratio varies with the structural organization and properties of the thick filaments, such as the length and the maximum active tension [11].

There was one contig of 13 ESTs and five singletons showing homology to paramyosins. With the addition of 3'ESTs of selected clones, the assembly produced a contig encoding a peptide with 77% identity to the N-terminal 138 aa of cattle tick paramyosin, while the remaining contigs and singletons encoded peptides with 64–70% identity to different parts of cattle tick paramyosin. Alignment of the translated peptides revealed the existence of at least two isoforms in regions homologous to both N and C terminals of paramyosin. The identities between the isoforms were 71% and 76% in each terminal. The putative paramyosins in tarantula showed 52–65% identity to *Drosophila melanogaster *paramyosin.

#### Actin

As the main component of muscle thin filaments, actin is reported to comprise 10 percent by weight of the total cell protein in muscles. It is also abundant in non-muscle cells, making up 1–5 percent of the cellular proteins. Actin is one of the most conserved proteins and is encoded by a large gene family. In invertebrates, muscle actins are typically most similar to vertebrate cytoplasmic actins [3].

There were 4 contigs and a few singletons, with 345 ESTs (14% of the total) showing homology to actin. The two major contigs were composed of 276 and 58 ESTs each. The translated sequences of these contigs and singletons were of two kinds. One was a complete propeptide of 376 aa encoded by the most abundant contig (Figure S3, Additional File 3), while the other (encoded by a contig of 4 ESTs) lacked the N-terminal 27 aa. These were designated as ACT1_As and ACT2_As respectively. There were 14 substitutions between the two actin isoforms, five of which were nonconservative (Figure S4, Additional File 4).

ACT1_As had 93–96% identity to various invertebrate actins, while ACT2_As showed 95–99% identity. Both isoforms were more similar to cytoplasmic actins than to muscle actins in *Drosophila *(Figure S4, Additional File 4), although the similarity to both was high. It has been reported that insect muscle including both non-flight and flight muscle actins form a distinct family of closely related proteins characterized by the presence of about 10 muscle-specific residues, including Asp (3), Ile (76), Ala (232), Thr (234), Val (278), Ile (325) and Gly (368) in mature actins [42]. Of these, only Asp (3) and Ile (76) were identified in the two tarantula isoforms. Given that the ESTs encoding ACT1_As are much more prevalent than ACT2_As, we assume that ACT1_As is the muscle isoform. Analysis of public ESTs from hemocytes of another tarantula, *Acanthoscurria gomesiana *showed 3 full-length actin propeptides, among which one is identical to ACT2_As except for one substitution, and all were more similar to ACT2_As than ACT1_As. Since the actins expressed in the hemocyte are presumably cytoplasmic isoforms, this supports the conclusion that ACT1_As is the muscle isoform while ACT2_As is the cytoplasmic isoform in *Aphonopelma*. Tarantula and insects belong to different subphyla of the arthropods (Chelicerata and Hexapoda, respectively). Our study thus shows that muscle actins might differ between these subphyla. Since there is a lack of muscle actins from chelicerates in public databases, the muscle-specific residues in chelicerate actins and their origins await further study of more species.

The positions that affect the binding of ATP (residues Lys214, Glu215, Gly303, Tyr307 and Lys337) and Ca^2+ ^(residues Asp12, Lys19, Gln138 and Asp155) to actin are conserved in both tarantula actin isoforms compared with other species[43] (Figure S4, Additional File 4). Two nuclear export sequences (NES-1, residues 171–182; NES-2, residues 212–223 in the tarantula actins) were identified [44].

#### The troponin-tropomyosin complex

In most muscles contraction is regulated in part by the complex of troponin and tropomyosin, which binds periodically to the actin backbone of the thin filament [45]. Tropomyosin (Tm/TPM) is an α-helical coiled-coil dimer, about 40 nm long, lying head-to-tail along each of the two long-pitch actin helical strands. Each tropomyosin interacts with seven actin monomers and binds one troponin complex, consisting of one copy each of troponin C (TnC), troponin I (TnI) and troponin T (TnT). Each Tm.Tn complex regulates seven actin monomers, by allowing or inhibiting the binding of myosin heads to the actin subunits [45]. The Tn components receive their identifying letter from their first identified property. TnC belongs to a large family of Ca^2+ ^binding proteins. On muscle stimulation, the binding of calcium to TnC initiates a cascade of conformational changes in Tm.Tn, abolishing inhibition by the complex, and allowing the formation of cross-bridges between actin and myosin heads. Troponin I inhibits actomyosin ATPase when bound one-to-one to actin. Troponin I consists of 181–211 amino acid residues, and has a calculated pI of approximately 9.9 due to an excess of positively charged residues. Troponin T is the tropomyosin-binding subunit of troponin. It also interacts with the N-terminus of TnI/C-terminus of TnC via its C-terminus, resulting in the tripartite structure that is critical for Ca^2+ ^activation of muscle [46].

#### Troponin C

Two contigs encoding TnC homologues were found. One consisted of 15 clones and contained a complete open-reading frame. The deduced peptide was designated as TNNC1_As. The other consisted of 3 clones, and encoded the C-terminal 46 aa exactly the same as TNNC1_As. However, the 3'UTR had several insertions, indicating that there are two transcriptional isoforms of TnC (Figure S5, Additional File 3).

TNNC1_As was 152 aa in length, and had no tryptophans or cysteines, in common with other arthropod TnCs, and in contrast to scallop TnCs [47]. It also had no proline, but 2 tyrosines. It showed the highest homology to TnC in Japanese horseshoe crab (86%), a close evolutionary relation, and relatively lower homology to TnCs in most invertebrates (55–70%), scallops and vertebrates (about 40%), and calmodulins (45–48%).

Structural studies have shown that TnCs are dumbbell-shaped proteins composed of two globular domains, with a pair of EF hands in each, connected by an α-helical linker. The four EF hands, labelled sequentially as sites I-IV, each comprise a helix-loop-helix structural motif, where Ca^2+ ^can potentially be bound by oxygen ligands of 6 amino acids in the loop region at positions 1, 3, 5, 7, 9 and 12 [36]. We compared TNNC1_As to the Ca^2+^-coordination consensus sequence and to *Drosophila *and crab TnCs. Based on sequence analysis, sites I and III appear to be abortive Ca^2+^-binding sites due to non-conservative amino acid replacements at the key Ca^2+^-coordinating positions, while sites II and IV are most likely to be active Ca^2+^-binding sites, as in other arthropods (Figure S5, Additional File 4). Consistent with previous findings in invertebrates, the C terminal half of TNNC1_As is more conserved than the N terminal half, and the Ca^2+^-binding loop of site IV is the most highly conserved [48]. Ca^2+ ^binding studies are needed to reveal the affinity of sites II and IV for Ca^2+^.

#### Troponin I

There were five clusters homologous to TnI in the present study, composed of 94, 54, 12, 5 and 1 clones each. The two largest clusters shared the same coding region of 148 aa in the C-terminus, but only the first cluster contained a complete ORF for 206 aa.

Although the second cluster was the longest, it yielded only partial coding sequence. The putative peptides from the remaining clusters were different. The cluster of 5 clones contained complete ORF for 199 aa, and the one of 12 clones encoded a 167 aa long peptide, lacking the C-terminal coding region. We conclude that there are at least 4 TnI isoforms in tarantula skeletal muscle, and designate the 206 aa putative full-length peptide as TNNI1_As (Figure S6, Additional File 3)–the tarantula major TnI isoform, the 199 aa putative full-length peptide (Figure S7, Additional File 3) as TNNI2_As, and the 167 aa putative partial peptide as TNNI3_As.

Ascidians and *Drosophila *produce long and short TnI isoforms (the longer isoforms containing a proline-rich block of extra sequence near the N-terminus) by an alternative RNA splicing mechanism from a single gene [49]. All of the three tarantula TnIs lack the N-terminal proline-rich extension of about 64 aa in alignment with the *Drosophila *long TnI and seem to fall instead in the category of the short isoforms of TnI. TNNI1_As and TNNI3_As share 91% identity, with all of the different amino acids scattered in the region of 90–123 aa (Figure S6, Additional File 4). In comparison with TNNI2_As, TNNI1_As shows 65% identity and TNNI3_As 71% identity, and there are deletions of Gln4, Ser5, Gly38 and an insertion of Tyr153 in both TNNI1_As and TNNI3_As. Regions of conservation observed in alignment of the three tarantula TnIs suggest that they are likely to be alternatively spliced products of a single gene (Figure S6, Additional File 4). When searched against Swiss-Prot database, TNNI1_As shows the highest homology to TnI in *Drosophila melanogaster *(56% identity), and relatively lower homology to TnIs in other invertebrates (43–56%) and vertebrates (about 27–34%). TNNI2_As and TNNI3_As show 61% and 62% identity each with TnI in *Drosophila melanogaster*.

**Figure 5:**
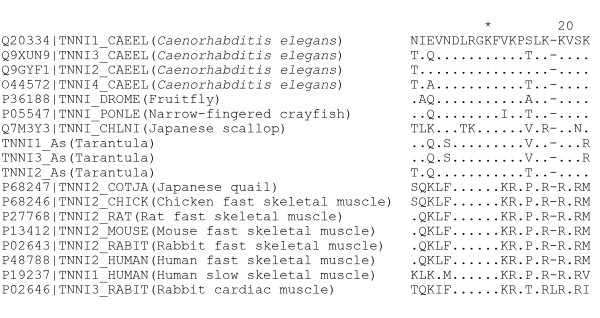
**Alignment of the minimal inhibitory peptide region of the tarantula TnIs to known TnIs**. The Swiss-Prot accession number (if available), abbreviation and source of each TnI are shown from left to right before the sequence. Dot indicates identity with the top sequence; Dash indicates a gap inserted for spacing purposes.

A minimal/main inhibitory peptide region in TnI has been reported to interact with actin and troponin C, and retain the important properties of intact TnI such as inhibiting actomyosin ATPase activity. The region in rabbit fast skeletal muscle consists of residues 96–116, 96NQKLFDLRGKFKRPPLRRVRM116 [46]. Multiple sequence alignment of TnI from both invertebrates and vertebrates shows that this region is also more conserved across species than other regions, and is highly similar within invertebrates or vertebrates, yet clearly different between them. The corresponding inhibitory region in TNNI2_As is identical to TNNI3_CAEEL, while the corresponding regions in both TNNI1_As and TNNI3_As are identical and have 4 different residues compared with TNNI2_As (Figure 5). The high similarity of these regions in the tarantula TnI isoforms to the invertebrate inhibitory region clearly establishes their identity as TnI.

#### Troponin T

Two contigs and four singletons encoding TnT homologues were found. The most abundant contig consisted of ESTs from 58 clones and contained a complete open-reading frame for 351 aa, designated as TNNT1_As or the major isoform (Figure S8, Additional File 3). The other consisted of ESTs from 9 clones, and encoded the C-terminal 157 aa, designated as TNNT2_As. The coding regions of the two contigs showed 94% identity, with 6 conservative substitutions and 3 non-conservative substitutions. The coding regions of two singletons showed 100% identity and those of the two other singletons only 57% identity to the N-terminal of TNNT1_As. We conclude that there are at least two isoforms of TnT in tarantula skeletal muscle.

In tarantula, the major TnT is less conserved than TnC and TnI. It showed the highest homology to TnT in the American cockroach (52% identity), 51% identity to TnT in *Drosophila melanogaster*, and relatively lower homology to TnTs in vertebrate skeletal muscle (about 31–35% identity).

In arthropods such as *Drosophila*, a negatively charged, Glu-rich C-terminal extension compared with vertebrates has been proposed to bind Ca^2+ ^and influence Ca^2+ ^activation of the muscle [50]. The tarantula major TnT also has a Glu-rich C-terminal extension, though shorter than in *Drosophila*. A special feature is that it contains four consecutive prolines and is Alanine-Proline rich. The tarantula major TnT lacked the N-terminal of more than 46 aa in alignment with TnTs from other invertebrates and vertebrates (Figure S7, Additional File 4). Since there are several stop codons and polyadenylation signals in the 5'-UTR of 380 nucleotides of the major TnT cDNA (Figure S8, Additional File 3), it seems that the shorter N terminal in the tarantula major TnT is not due to improper conceptual translation. It has been reported that the N-terminal region of TnT is hypervariable and plays a role in fine-tuning contractility though it does not interact with TnC, TnI and Tm directly [51]. The authenticity and significance of the shorter N-terminal region of tarantula TnT would be an interesting point for further exploration. In alignment with other invertebrate TnTs, there are two long consecutive insertions and deletions in the tarantula major TnT, suggesting specific exons for alternative splicing (Figure S7, Additional File 4).

#### Tropomyosin

The tropomyosins comprise a family of actin filament binding proteins, important not only in muscle regulation, but in cytoskeletal function in nonmuscle cells. Tropomyosin isoforms provide a powerful mechanism to diversify actin filament function in different intracellular compartments [52].

Assembly of 33 of the tarantula ESTs produced a contig highly homologous to tropomyosin, containing a complete ORF. Its deduced peptide had 284 amino acid residues and a predicted mass of 33 kDa, consistent with a typical muscle tropomyosin. This was designated as TPM2_As (Figure S9, Additional File 3). Another contig composed of 65 clones encoded the C-terminal 176 amino acid residues and was designated as TPM1_As. Comparison of the two putative peptides showed 8 substitutions in total, all in the 214–234 aa region, which corresponds to the region encoded by exon 7a/7b of the tropomyosin I gene in *Drosophila melanogaster *[53], and indicates that they might be alternative splicing products from the same gene (Figure S8, Additional File 4). We conclude that there are at least 2 tropomyosin isoforms in tarantula skeletal muscle.

The full-length TPM2_As shows the highest homology to TPM in cattle tick (92%), 64–75% identity to TPMs in *Drosophila melanogaster*, and 50–59% identity to TPMs in vertebrates. Like conventional muscle tropomyosins, both tarantula isoforms are predicted to be coiled-coil over their lengths and lack the Pro-Ala-Gly-Glu rich C-terminal present in high molecular weight tropomyosins specific to the indirect flight muscle of *Drosophila melanogaster *[54].

### Implications of protein isoforms in tarantula skeletal muscle

This is the first time that multiple transcriptional isoforms of most myofibrillar proteins have been identified in tarantula (Table 1). Post-translational modification, such as phosphorylation, might further increase the number of isoforms. The isoform diversity revealed in our study confirms the generality of the phenomenon in skeletal muscle and emphasizes the need to understand its importance in muscle function.

**Table 1:** Transcriptional isoforms of tarantula muscle filament proteins.

**Abbreviation**	**CDS**	**Length(AA)**	**#Clones**	**pI**	**Mw (Da)**
ACT1_As	complete	376	276	5.29	41826.83

ACT2_As	partial, lack N-terminal	349	58	\	\

TNNC1_As	complete	152	18	4.09	17518.55

TNNI1_As	complete	206	148	9.7	23616.27

TNNI2_As	complete	199	5	\	\

TNNI3_As	partial, lack C-terminal	167	12	9.84	19329.5

TNNT1_As	complete	365	67	5.39	43296.7

TNNT2_As	partial, lack N-terminal	157	4	\	\

TPM1_As	partial, lack N-terminal	176	33	\	\

TPM2_As	complete	284	65	4.65	32992.72

MLE1_As	complete	156	12	4.62	17603.09

MLE2_As	partial, lack N-terminal	87	12	\	\

MLR1_As	complete	196	125	4.74	21765.3

MLR2_As	complete	235	5	4.66	26054.29

MYHhead1_As	partial	620	PCR product	\	\

MYHhead2_As	partial	310	3	\	\

MYHhead3_As	partial	938	20	\	\

MYHhead4_As	partial, with N-terminal	268	1	\	\

MYHtail1_As	partial, with C-terminal	391	115	\	\

MYHtail2_As	partial	721	11	\	\

The abbreviations of tarantula muscle filament protein transcriptional isoforms, the completeness of their coding sequences (CDS), the lengths, the number of clones comprising the isoforms, and the predicted pI and Mw are shown.

The existence of multiple isoforms of myofibrillar proteins may be related to a mix of fiber types in tarantula skeletal muscle. It has been reported that different muscle fiber types (e.g. fast and slow fibers in vertebrates) express different specific spectra of myofibrillar protein isoforms [55-57]. Though muscle fiber types in tarantula leg muscle have not been investigated, the overall structure and biochemistry of tarantula fibers is similar to that of *Limulus*, another chelicerate, which shows at least two fiber types [58]. In addition, hybrid fibers which exist as points on a continuum of fiber types and possess a phenotype that is intermediate between the extremes of pure/distinct fiber types, seem to be prevalent in many muscles [55-57]. For example, there have been reports that a single fiber from lobster expresses a mixture of myofibrillar mRNA and protein isoforms [55]. It is therefore possible that there are a substantial number of fiber types rather than just the few according to the traditional classification in skeletal muscle. The correlation between myofibrillar protein isoform and putative muscle fiber type is yet to be understood in tarantula, as is the role of each isoform.

EST counts have been reported to reflect the abundance of the corresponding transcripts in the tissue or cell from which the standard cDNA library was constructed [59]. Their numbers might therefore be expected to reflect the approximate molar proportions of the corresponding proteins in that tissue or cell. The molar ratio of MHC: ELC: RLC in myosin II is 1:1:1 [38], while the ratios of troponin C: I: T and actin: tropomyosin: troponin are 1:1:1 and 7:1:1 in thin filaments [45]. It has also been reported that the paramyosin: myosin heavy chain molar ratio is ~0.3 in tarantula muscle [12]. In contrast, summation of EST counts from all different isoforms of these proteins gives EST ratios of: ELC: RLC (26:124), ACT: TPM: Tn (345:249:98), TnC:I:T (18:160:71) and PM:MHC (18:8), which differ substantially from the protein stoichiometries. A possible explanation for the discrepancy is that only one or a few, instead of all, isoforms of myofibrillar proteins participate in muscle filament assembly. Furthermore, estimation of mRNA/transcript abundance from EST counts is affected by sampling size and random fluctuations [59]. Statistical analysis [59] shows that the mRNA abundances among TNNC1_As, TNNI2/3_As, TNNT2_As might not be significantly different, and it could be possible that they form Tn with a 1:1:1 TnC:I: T ratio. Similarly, ACT1_As, TNNC1_As, TNNI2/3_As, TNNT2_As together with TPM1_As could possibly form 7:1:1 complex as found in the thin filament. However, even after statistical consideration, there is no ELC with abundance comparable to the major isoforms of RLC and MHC, and likewise no TnC comparable in abundance to the major isoforms of TnI, TnT and TPM. Possible explanations for these remaining discrepancies are differences in posttranscriptional regulation, translational efficiency, protein turnover rate, and limitations in the different techniques [60,61]. Since proper stoichiometry is essential for muscle filament assembly, the expression level of different isoforms of each myofibrillar protein deserves further investigation using quantitative expression analysis. It would be interesting to explore which isoforms of MHC, ELC and RLC assemble into the hexameric myosin, which isoforms of troponin I and T together with C assemble into the troponin complex, and how troponin interacts further with isoforms of TPM and actin to form the thin filament.

## Conclusion

Through the generation and analysis of ESTs from tarantula skeletal muscle, our study identified transcriptional isoforms of the major contractile filament proteins as well as homologues of muscle LIM Protein, muscle protein 20, paramyosin, α-actinin, MLCK, calponin, and Ig/Fn motifs (found in giant sarcomere-related proteins). This will serve not only as a basis for defining the transcriptome of tarantula skeletal muscle, but also as an aid in understanding the molecular biology of the muscle proteins and the mechanism of contraction in the model tarantula system and in skeletal muscles in general. The cDNA clones and their analysis will also provide sources and ideas for further experimental characterization of tarantula muscle proteins and filament assembly.

## Methods

### Tarantula skeletal muscle sampling

Adult tarantulas *Aphonopelma *sp. were purchased from Carolina Biological Supply Co. (Catalog#:14-3350), USA. They were chilled at -20°C for 10–15 min, and then the leg muscles were dissected on ice and immediately placed in Eppendorf tubes in liquid nitrogen until RNA extraction.

### cDNA library construction

Total RNA was isolated from 9 grams of tarantula skeletal muscle using TriZol reagent (Invitrogen), quantified in a spectrophotometer, and the quality verified by agarose gel electrophoresis. mRNA was obtained using Oligotex mRNA isolation kits (QIAGEN). Double-stranded cDNA was synthesized with oligo-dT primer. cDNAs larger than 500 bp were cloned into pBluescript^® ^II XR Predigested Vector (Stratagene) and transformed into *Escherichia coli *DH10B competent cells (Invitrogen). The average insert length of the cDNA library was 1–2 kb. The titer of the library was 4.0 × 10^5 ^cfu/ml.

### EST sequencing and bioinformatic analyses

Clones containing inserts were randomly picked and cultured overnight. The plasmids were extracted according to a standard alkaline lysis protocol. Single-pass sequencing of the 5'-termini was conducted with standard T3 primers using an ABI3730 automated sequencer (Applied Biosystems) according to the manufacturer's instructions.

The chromatogram files were processed as raw data for base-calling and quality assessing by Phred software (Phred-Phrap-Consed package) [62]. The low-quality sequences were trimmed off with Q20 (99% accuracy). The vector sequences were screened with the program CROSS_MATCH. Sequences containing more than 100 bp after quality and vector trimming were regarded as high quality sequences, which were then assembled by CAP3 software using default settings (overlap length cutoff > 30 and overlap percent identity cutoff > 75) [63]. The resulting contigs and singletons were inspected by Consed software [64]. (A contig is the DNA sequence reconstructed from a set of ESTs with significant overlaps. A singleton is an EST without any significant overlap with the other ESTs. The contigs and the singletons were all called clusters.)

All clusters were searched against the nr protein database in GenBank and Swiss-Prot by BLASTX with e-values 1e-5 to identify homologues and assign possible function [65]. The clusters without hit to nr were searched against the nt nucleotide database in GenBank by BLASTN with e-values < 10^-10 ^and the remaining clusters without hit to nt were searched against the Chelicerate ESTs in dbEST by BLASTN with e-values < 10^-10^. The clusters without hits to Swiss-Prot were searched against the TrEMBL database with e-values < 10^-5^. Clusters with the same best hit from similarity searches are likely to come from the same transcript, and were thus grouped as a unigene.

A cluster is designated as full-length if it contains a complete open reading frame (ORF), established by BLAST similarities [66], and portions of the 5' and 3' untranslated regions (UTRs), indicated by start and stop codons as well as polyadenylation signals. Putative peptides were obtained by conceptual translation with OrfPredictor [67], then searched against protein databases with BLASTP for verification and modification [65].

The tarantula *Acanthoscurria gomesiana *haemocyte public ESTs [9] were retrieved from dbEST and processed as above.

### Increasing cDNA coverage

Some clones of clusters with incomplete coding regions, yet annotated as skeletal-muscle specific or enriched transcripts, were selected to be sequenced from 3' ends using T7 primers. Significant clones were subjected to further sequencing by primer walking to get the full insert of the clones after both 5' and 3' EST sequencing. The sequences were processed as above except with phred Q13 and CAP3 parameters set to overlap 40 and identity 90 in the assembly with corresponding ESTs. All the sequences including 5' and 3' ESTs and extended sequences were assembled again finally using CAP3 with overlap 40 and identity 90 and then annotated for verification. Primer-specific RT-PCR was also performed to bridge the gap in the coding sequences.

### Protein Analysis

Domain analysis was performed by InterProScan searches against the InterPro database [68,69]. Multiple sequence alignments were carried out using MUSCLE [70] or CLUSTAL_X [71]. Protein physiochemical property analysis was carried out using ExPASy webserver [72]. Phosphorylation site prediction was done with Group-based Phosphorylation Scoring method [28].

## Authors' contributions

JGZ analyzed the EST data, designed the experiments for increasing cDNA coverage and wrote the initial draft of the manuscript. YQS carried out the cDNA library construction, preparation of the clones for sequencing and RT-PCR. FQZ initiated the project and made tarantula leg muscle preparation. JY participated in the design of the project. RC and SNH supervised the entire project and revised the final version of the manuscript. All authors read and approved the manuscript.

## Supplementary Material

Additional file 1 **dbEST submission. **Complete submission to the dbEST database. Click here for file

Additional file 2 **Functional annotation of the 5'ESTs. **Functional annotation of the 5'ESTs based on Nr matches, Uniprot (SwissProt and TrEMBL) matches, Nt matches and dbEST matches. Click here for file

Additional file 3 **Supplementary figures (sequences). **The nucleotide and deduced amino acid sequences of putative full-length major muscle proteins are shown. Stop codons and putative polyadenylation signals are underlined. Click here for file

Additional file 4 **Supplementary figures (sequence alignments).** The alignments of tarantula myofibrillar proteins with other species. Click here for file
