# Supplementary material for: Analysis of tarantula skeletal muscle protein sequences and identification of transcriptional isoforms
Source: BMC Genomics. 2009 Mar 19;10:117. doi: 10.1186/1471-2164-10-117 (PMC2674065; doi:10.1186/1471-2164-10-117)
Supplement: Additional file 3 — Supplementary figures (sequences). The nucleotide and deduced amino acid sequences of putative full-length major muscle proteins are shown. Stop codons and putative polyadenylation signals are underlined. [file 1471-2164-10-117-S3.pdf]

```
   1 CCCCCGGGCTGCAGGAATTCGAATTCCCTCGACTTGCACTTGCGGTTTAGGTCGGTTTTG 60

  61 TGTTCTAAAGACAGGAATAatgggtgacgaggagaagaaagagaagaagaagaagagcaa 120
   1                    M  G  D  E  E  K  K  E  K  K  K  K  S  K  14
 121 gaagaagtcagaggaagagggtggagatgccgcccctgcacctcccccacctaagccccc 180
  15  K  K  S  E  E  E  G  G  D  A  A  P  A  P  P  P  P  K  P  P  34
 181 gtcccagaaaaggagagcacaaagatcaggcagtaatgtctttgccatgttcactcagca 240
  35  S  Q  K  R  R  A  Q  R  S  G  S  N  V  F  A  M  F  T  Q  H  54
 241 ccaggtccaggagttcaaagaagctttccaacttattgaccaggacaaggatggtttcat 300
  55  Q  V  Q  E  F  K  E  A  F  Q  L  I  D  Q  D  K  D  G  F  I  74
 301 ctccaaaaatgacatcagagctacattcgattcccttggccgtctatgcacagagcagga 360
  75  S  K  N  D  I  R  A  T  F  D  S  L  G  R  L  C  T  E  Q  E  94
 361 gcttgattccatggttgctgaagcacccggccccatcaacttcaccatgttcctgaccat 420
  95  L  D  S  M  V  A  E  A  P  G  P  I  N  F  T  M  F  L  T  I  114
 421 cttcggtgacagaattgctggaaccgatgaagaagatgtgattgttaacgctttcaacct 480
 115  F  G  D  R  I  A  G  T  D  E  E  D  V  I  V  N  A  F  N  L  134
 481 gtttgatgaaggtgaaggcaaatgcaaagaagaaacattgaaacgttctctaacaacatg 540
 135  F  D  E  G  E  G  K  C  K  E  E  T  L  K  R  S  L  T  T  W  154
 541 gggagagaaattctctcaagatgaggttgaggaagccctgtctgaagctcctattgatgg 600
 155  G  E  K  F  S  Q  D  E  V  E  E  A  L  S  E  A  P  I  D  G  174
 601 aaacggcttgattgacatcaagaagtttgctcagatcttaactaagggtgcagaagaaga 660
 175  N  G  L  I  D  I  K  K  F  A  Q  I  L  T  K  G  A  E  E  E  194

 661 gggagctTAAATTACTCGTCATCTACGGCATCCATTACCAAGGTTGGAATCTTGTGACCT 720
 195  G  A                                                         196
 721 GCAGGATTCTCTAGAAATCAAAGAACTACGCATATTTCATTCTTCCAAGCAAAGACCACT 780

 781 GTAAATAGAAAGGACTACAGTTACTGTCACCCGTAGCCTCATGTGAACACCTGTCTTTCA 840

 841 AAGGAAGTGTATCTAGCAGTGGACTTTTTTTTTTCTTGCTTTCACCTAGTGGTCACAAGA 900

 901 TGATGCCTACCTTGGAAACCGTTGTTTTGATGTCCATTTTCTCTCTGAAATCTATCTTCA 960

 961 TCTTTCGTGGGTGATGTGAAATCTGAAAAGAAATGGACGTTGTCAGTATTTTCCTCGTCA 1020

1021 TGTTCAAATTTCTACGTCAAAATCAAAAAACTTTTTATTTGTTAAATCTGCGGCCGCACT 1080

1081 GATGAAAATCAATTACCCTGAGAATTTTGCTTCAAACATTGGCATTTTACATTTGTTTAA 1140

1141 ATTATTATTAACATCTATTGGTCTCAAATTGAAATAAATATGTCGTGTACTGAAATTTGA 1200

1201 CAGAAAAAGTGTAAATGACTCGTTTTTATTTAATGTATTATTTATTTGGACCGAATTAAC 1260

1261 CATGAATGTAATTCTGCATATCATCTGTTTGTACATTTTTAAAAGAGATTAAGTATTCGC 1320

1321 GTTACAACTTGGGCCTGGTAGGGCTTGCCTGCCTTGTAAGAAAGCCAGTGCTTTTCTTAC 1380

1381 TGGAAAAGTACGCATGAATAAAGTTAAATTTTCAAAAAAAAAAAAAAAAAAAAAAAAAAA 1440

1441 AAAAAAAAAAAAAAT 1455
```

Supplementary Figure 1 - Nucleotide and predicted amino acid sequences of MLR1_As.

```
   1 ATCCCCCGGGCTGCAGGGTGGTGCCCGTGAAGAGCTTCCGTGAGCATCAGGAGCCAGGGA 60

  61 GGAGGAAAGTTGGTTTTGAGTTGGATTTTCCTGCATCCTAatggcagaagaggagaagaa 120
   1                                         M  A  E  E  E  K  K  7
 121 agagaaaaagaagaaaagcaaaaagaaggcagaagctgaagcagctccagcaccacctcc 180
   8  E  K  K  K  K  S  K  K  K  A  E  A  E  A  A  P  A  P  P  P  27
 181 ggaagcagccccggcagaggaagctccaccagcggcggcagaacctgtgtctgaaccagt 240
  28  E  A  A  P  A  E  E  A  P  P  A  A  A  E  P  V  S  E  P  V  47
 241 agtagactcagttcctgtggacgaggctcctccacctgaaccacctgcaccaacaccagc 300
  48  V  D  S  V  P  V  D  E  A  P  P  P  E  P  P  A  P  T  P  A  67
 301 gaaaaaagcatcgtcgaagaagagagcccagagatcgggatccaatgttttcacgatgtt 360
  68  K  K  A  S  S  K  K  R  A  Q  R  S  G  S  N  V  F  T  M  F  87
 361 cacacaaaggaaggttcaagaattcaaagaggctttccagctcatagaccaagacaagga 420
  88  T  Q  R  K  V  Q  E  F  K  E  A  F  Q  L  I  D  Q  D  K  D  107
```

```
 421 cggattcatcacaaagtctgatctcaaaataacgtttgatctgttgggtcgtgatgttga 480
 108  G  F  I  T  K  S  D  L  K  I  T  F  D  L  L  G  R  D  V  D  127
 481 tgacgaagatttacaagatatgttggccgaagcccctggtccattgaatttcaccatgtt 540
 128  D  E  D  L  Q  D  M  L  A  E  A  P  G  P  L  N  F  T  M  F  147
 541 tttaacaatatttggtgaaagaatttcggggactgatgaagaagatgtgatcttaaacgc 600
 148  L  T  I  F  G  E  R  I  S  G  T  D  E  E  D  V  I  L  N  A  167
 601 cttccaaattttcgacgaaggagaaggaaaaatgaaagaggaagtgtttaaaaacaccct 660
 168  F  Q  I  F  D  E  G  E  G  K  M  K  E  E  V  F  K  N  T  L  187
 661 aaagaagagaggggataagttcactccagaagaagcggacatctgtctgaaggaagctcc 720
 188  K  K  R  G  D  K  F  T  P  E  E  A  D  I  C  L  K  E  A  P  207
 721 tgtggataaagaaggatatatcagtattcgtcggtttacaagaataatcactaagggtga 780
 208  V  D  K  E  G  Y  I  S  I  R  R  F  T  R  I  I  T  K  G  E  227

 781 ggaagaagatgacgcagatggaagtTAAAGGTGCGCCTTCTACTTTAATAGTTTGATGAG 840
 228  E  E  D  D  A  D  G  S                                       235
 841 AAATAGTAGACCGGATACAGTTCTGAGATTTCCTGCTCGAAACAAAAGAACAGAGAACTG 900

 901 TTCCTCAAGAAGACACTACTTAAAATATGCAAAGAAGTACCGAACTTTCTACAGAGTTTA 960

 961 ACAAAAACGGAATCTCAGATGTCTCATTTTCTAACATAGAATGCTTTTGTAAACTGATTC 1020

1021 ATTCTGAACTTGGGAAAAATAAAAACAGCCCTGCCTGGAACTATAAATAAAATGTTTCTG 1080

1081 TGTAATGTCACATGATTGCATTATTCCATATTTGTAACCAATAAAGTCATTCTGAACCAT 1140

1141 ATAGTGACAAAAAAACCTGCAGC 1163
```

Supplementary Figure 2 - Nucleotide and predicted amino acid sequences of MLR2_As.

```
   1 AAAAAAAAAAAAAACTCGGCTCGTTTGTTGTGTAGTTGCGTACTGTGCGGACATAGTCAG 60

  61 GAATatggcagacctcaaggccgctgaagttgaaaaggcaagggagcactttgagatcta 120
   1     M  A  D  L  K  A  A  E  V  E  K  A  R  E  H  F  E  I  Y  19
 121 tgactgggaaggcgagggcaaaatcgatgccagagatctgggtgatctcttgaggtcgct 180
  20  D  W  E  G  E  G  K  I  D  A  R  D  L  G  D  L  L  R  S  L  39
 181 ggactgcaagccaacactggctatggtaaaaaagaacggcggttccgacaaaagaggcga 240
  40  D  C  K  P  T  L  A  M  V  K  K  N  G  G  S  D  K  R  G  E  59
 241 aaagaaattaacgctagaggaattcctacctatcttcagccaaatcaaaaaagaaaaaga 300
  60  K  K  L  T  L  E  E  F  L  P  I  F  S  Q  I  K  K  E  K  E  79
 301 agttggaactctagaggacttcatggagggacttaaagtttacgacaaagcagaaaacgg 360
  80  V  G  T  L  E  D  F  M  E  G  L  K  V  Y  D  K  A  E  N  G  99
 361 aaccatgttagctgctgaattagcacacgtattgctctcattaggtgaaagattaacaga 420
 100  T  M  L  A  A  E  L  A  H  V  L  L  S  L  G  E  R  L  T  D  119
 421 catagaatgtgaagaaatcatgagggtctgcgacgaagatgatgacggattcctcaaata 480
 120  I  E  C  E  E  I  M  R  V  C  D  E  D  D  D  G  F  L  K  Y  139
 481 cgaacctttgtcaagaccattatagcagggccattcccagacgagggtaaaTGAACAGG 540
 140  E  P  F  V  K  T  I  I  A  G  P  F  P  D  E  G  K           156
 541 AGCATTTTAAGCACCTTTTTTTTATGTAGAAAAATTTCAAGTTCGGATATGACTACTATT 600

 601 TGTTCTACTGGGTCATCTTTTAATGAAAACTACTTGTTTGTAAATTTCTGTGATTGATGT 660

 661 AAAAAACCAACTCATCAGTCCATTAAGCAACTGATCTCATGATTATTGGCAATTATATTT 720

 721 TGAACCTGAAAAACAACTAAATATTCTACATTTTTTCAGTCGGCCATTAAAAAAAAAAAC 780

 781 GACAAAAATATTCTATGTGTTTATGAGTCATTCAAACTAAAAATAAATTTCCTTTCTGCC 840

 841 AACAAAAAAAAAAAAAAAAA 860
```

Supplementary Figure 3 - Nucleotide and predicted amino acid sequences of MLE1_As.

```
   1 GGATCCCCCGGGCTGCAGGAATTCGAATTCCTTCCATTCCATCGGATCTATTCCGGATCT 60

  61 CTTCGTGCGTTCCCAGGAAAAAACCGTAACAGTCCAGAatgtgtgacgacgacattgccg 120
   1                                       M  C  D  D  D  I  A  A 8
```

```
 121 cacttgttgttgacaatggctctggcatgtgcaaggccgggttcgccggagatgacgctc 180
   9  L  V  V  D  N  G  S  G  M  C  K  A  G  F  A  G  D  D  A  P 28
 181 ccagggctgtcttcccctccatcgtcggccgtcccaggcatcagggtgtcatggtgggta 240
  29  R  A  V  F  P  S  I  V  G  R  P  R  H  Q  G  V  M  V  G  M 48
 241 tgggtcaaaaggacagctacgtaggtgatgaggcccagagcaagagaggtatcctctccc 300
  49  G  Q  K  D  S  Y  V  G  D  E  A  Q  S  K  R  G  I  L  S  L 68
 301 tgaagtaccccatcgagcacggcatcatcactaactgggacgacatggagaagatctggc 360
  69  K  Y  P  I  E  H  G  I  I  T  N  W  D  D  M  E  K  I  W  H 88
 361 atcacaccttctacaacgagctccgagttgcccccgaggagcaccccatccttctcaccg 420
  89  H  T  F  Y  N  E  L  R  V  A  P  E  E  H  P  I  L  L  T  E 108
 421 aagctccccttaaccccaaggctaacagggaaaagatgacccagatcatgtttgagacct 480
 109  A  P  L  N  P  K  A  N  R  E  K  M  T  Q  I  M  F  E  T  F 128
 481 tcaacgcccccgccatgtacgttgccatccaggccgtactgtccctgtacgcttccggta 540
 129  N  A  P  A  M  Y  V  A  I  Q  A  V  L  S  L  Y  A  S  G  R 148
 541 ggaccaccggtattgtgctcgactccggtgatggtgtttcccacactgtacccatctatg 600
 149  T  T  G  I  V  L  D  S  G  D  G  V  S  H  T  V  P  I  Y  E 168
 601 aaggttacgctcttccccatgccattctccgtctggacttggctggccgtgatctgactg 660
 169  G  Y  A  L  P  H  A  I  L  R  L  D  L  A  G  R  D  L  T  D 188
 661 actacctcatgaagatcttgactgagaggggctactctttcgtaaccacagctgagcgag 720
 189  Y  L  M  K  I  L  T  E  R  G  Y  S  F  V  T  T  A  E  R  E 208
 721 aaatcgttcgtgacatcaaggaaaagctgtgctacgtcgccctggacttcgagcaggaga 780
 209  I  V  R  D  I  K  E  K  L  C  Y  V  A  L  D  F  E  Q  E  M 228
 781 tggccactgccgcttcctcctccacagtagagaagtcctacgagttgcctgacggtcagg 840
 229  A  T  A  A  S  S  S  T  V  E  K  S  Y  E  L  P  D  G  Q  V 248
 841 tcatcaccatcggcaatgagaggttccgttgccctgagaccctcttccagccttccttca 900
 249  I  T  I  G  N  E  R  F  R  C  P  E  T  L  F  Q  P  S  F  I 268
 901 taggtatggagtctgttggcatccacgagaccaccttcaactccatccagaagtgcgaca 960
 269  G  M  E  S  V  G  I  H  E  T  T  F  N  S  I  Q  K  C  D  I 288
 961 ttgatatccgtaaggatctgtacgccaacactgtcctgtccggtggcaccaccatgtacc 1020
 289  D  I  R  K  D  L  Y  A  N  T  V  L  S  G  G  T  T  M  Y  P 308
1021 ccggtattgctgacaggatgcagaaggaaatcactgctctcgctcccagcaccatgaaga 1080
 309  G  I  A  D  R  M  Q  K  E  I  T  A  L  A  P  S  T  M  K  I 328
1081 tcaagatcatcgctccccctgagaggaagtactccgtatggatcggtggctccatcttgg 1140
 329  K  I  I  A  P  P  E  R  K  Y  S  V  W  I  G  G  S  I  L  A 348
1141 cctcactgtccacattccagcagatgtggatctccaagcaagagtacgatgagtctggac 1200
 349  S  L  S  T  F  Q  Q  M  W  I  S  K  Q  E  Y  D  E  S  G  P 368
1201 ccagcattgtacatcgcaagtgcttcTAAACATCTGCTATTTATATCTTTAATGTTATTT 1260
 369  S  I  V  H  R  K  C  F                                     376
1261 TCTGGGTTACGTTTCGTGACTTTCAGCATCAACTGCCGTCTACTAAGATCAGACTGTACT 1320

1321 TGACAATAGCCCTGTTGCCGTAAAACGTGCTGAGCTAATAAAACCTAATCTGATCTCAGT 1380

1381 GCCTTACAGTCATCTGGCCACGAAATTTTATGTACAAACAAAGGCTGTTGTAATAAATAC 1440

1441 TTTTTGAGTACAAAAAAAAAAAAAAAAAAAAAAAAAAAAAAAAAAAAAAAAAAAAAAAAA 1500

1501 AAAAAAAAAAAAAAAAAAAAAAAAAAAAAAAAAA 1534
```

Supplementary Figure 4 - Nucleotide and predicted amino acid sequences of ACT1_As.

```
TNNC1_As.Contig  1    CGCTTCTCCGTTGTCAGTTTCCCTCGTCTCTTGCCGCAGACAATACCTACAGCCACACTT
TNNC_As.Contig2  1    ------------------------------------------------------------

TNNC1_As.Contig  61   CCACCATGGTTGAGGAGCTGAGCAAAGAGCAGGTTGAGATGTTGAAGAAAGCCTTCGACA
                            M  V  E  E  L  S  K  E  Q  V  E  M  L  K  K  A  F  D
TNNC_As.Contig2  1    ------------------------------------------------------------

TNNC1_As.Contig  121  TGTTCGACAGGGAGAAGAAAGGCTCCATCCACACCTCAATGGTATCAACAATCTTGAGAA
                      M  F  D  R  E  K  K  G  S  I  H  T  S  M  V  S  T  I  L  R
TNNC_As.Contig2  1    ------------------------------------------------------------

TNNC1_As.Contig  181  CTCTGGGGCAGACATTCGTGGAAAGTGAGCTAAAAGAACTAATTATAGAAATTGATCAGG
                      T  L  G  Q  T  F  V  E  S  E  L  K  E  L  I  I  E  I  D  Q
TNNC_As.Contig2  1    ------------------------------------------------------------

TNNC1_As.Contig  241  ACGGAAGCGGTGAGCTAGAATTCGATGAGTTCTTGGCACTAACAGCAAGGTTCTTGGTAG
                      D  G  S  G  E  L  E  F  D  E  F  L  A  L  T  A  R  F  L  V
TNNC_As.Contig2  1    ------------------------------------------------------------
```

```
TNNC1_As.Contig  301  AAGAGGATTCCGAGGCCATGCAAGAGGAACTTAGAGAAGCCTTCAGGATGTACGATAAAG
                      E  E  D  S  E  A  M  Q  E  E  L  R  E  A  F  R  M  Y  D  K
TNNC_As.Contig2  1    ------------------------------------------------------------

TNNC1_As.Contig  361  AAGGGAACGGGTACATCAACGTTTCGGACCTCCGAGAGATCCTCAGGGCTTTGGATGACA
                      E  G  N  G  Y  I  N  V  S  D  L  R  E  I  L  R  A  L  D  D
TNNC_As.Contig2  1    --------------------GTTTCGGACCTCCGAGAGATCCTCAGGGCTTTGGATGACA

TNNC1_As.Contig  421  AACTGACGGAAGATGAGCTTGACGAGATGATCGCTGAGATTGACACAGACGGTAGCGGAA
                      K  L  T  E  D  E  L  D  E  M  I  A  E  I  D  T  D  G  S  G
TNNC_As.Contig2  41   AACTGACGGAAGATGAGCTTGACGAGATGATCGCTGAGATTGACACAGACGGTAGCGGAA

TNNC1_As.Contig  481  CGGTAGACTTCGACGAGTTCATGGAGATGATGACTGGTGATTGAGCACTTTCTCTCCCTT
                      T  V  D  F  D  E  F  M  E  M  M  T  G  D
TNNC_As.Contig2  101  CGGTAGACTTCGACGAGTTCATGGAGATGATGACTGGTGATTGAGCACTTTCTCTCCCTT

TNNC1_As.Contig  541  GTGATCTACTGCTACGGGGGATATAAAACTACAACCACCTCTAGCTATAAAGGAATTCTC
TNNC_As.Contig2  161  GTGATCTACTGCTACGGGGGATATAAAACTACAACCACCTCTAGCTATAAAGGAATTCTC

TNNC1_As.Contig  601  GACCTTTATATTCTTTTTGG--------------CCTAAAAACTTCCATAAATAACATGT
TNNC_As.Contig2  221  GACCTTTATATTCTTTTTGGTTTAATTACACAGGCCTAAAAACTTCCATAAATAACATGT

TNNC1_As.Contig  647  ATAAATTCATATCAAAAAAGGGGGGAGGAAAACTCACAAGCCCAGAGGTACAATGTGAGA
TNNC_As.Contig2  281  ATAAATTCATATCAAAAAAGGGGGGAGGAAAACTCACAAGCCCAGAGGTACAATGTGAGA

TNNC1_As.Contig  707  GCATAACTTTTATGTAACCTGTTATTATTCGTTCTTATTTATGTTATTTTACGTTGAAGT
TNNC_As.Contig2  341  GCATAACTTTTATGTAACCTGTTATTATTCGTTCTTATTTATGTTATTTTACGTTGAAGT

TNNC1_As.Contig  767  TTTCGTCTGCAAGACGTCAGTTGTCGTTCTTTTTCGGTTAACTGTATAAAATGGCTGGTT
TNNC_As.Contig2  401  TTTCGTCTGCAAGACGTCAGTTGTCGTTCTTTTTCGGTTAACTGTATAAAATGGCTGGTT

TNNC1_Ah.Contig  827  CTAATAAAACTT-GGGGGGAATTATTTTCTCTGTCTCGTCTTTCTTTACGTTATCTTGTT
TNNC_Ah.Contig2  461  CTAATAAAACTTTGGGGGGAATTATTTTCTCTGTCTCGTCTTTCTTTACGTTATCTTGTT

TNNC1_As.Contig  886  TGCAAAAAGTGCCTAAAATCACATTTAGAATTTAGCGGAACATAGGTAGGAAAAAAAAGA
TNNC_As.Contig2  521  TGCAAAAAGTGCCTAAAATCACATTTAGAATTTAGCGGAACATAGGTAGGAAAAAAAAGA

TNNC1_As.Contig  946  GAGAGAGAGAGAGAGAAGGACCAGCTTTATGAAAATGGTTAATTGCAAAACTGTGTTTTA
TNNC_Ah.Contig2  581  GAGAGAGAGAGAGAGAAGGACCAGCTTTATGAAAATGGTTAATTGCAAAACTGTGTTTTA

TNNC1_As.Contig  1006 GTTCACATTACTTAGTTAAGGCGTAACATTTCATTCTTTCATCTTTTCACACTTGTGTTA
TNNC_As.Contig2  641  GTTCACATTACTTAGTTAAGGCGTAACATTTCATTCTTTCATCTTTTCACACTTGTGTTA


TNNC1_As.Contig  1066 AGTGATGTTCTTTATGCCATGTATGACAAATACAGAAAAAAATGAAAGTGTAAAAAAATG
TNNC_As.Contig2  701  AGTGATGTTCTTTATGCCATGTATGACAAATACAGAAAAAAATGAAAGTGTAAAAAAATG

TNNC1_As.Contig  1126 TAAATAATTTAATGAATGAATGAATAAATAAAT---------------------------
TNNC_As.Contig2  761  TAAATAATTTAATGAATGAATGAATAAATAAAT---------------------------
```

Supplementary Figure 5 - Nucleotide and predicted amino acid sequences of two TnC isoforms.

```
  1 ATTCCAGCTCCAATAGCGTATATTAAAGTTGTTGCGGTTAAAAAGCTCGTAGTTGGATCT 60

 61 CAGTTCCAGACGGGCGGTCCGCCTAACGGTGGTTACTGCCTGGCCTGAACAGCCAGCCGG 120

121 TTTCCCTAGATGATCTTCACCGGTTGTCCTGGGTGACCGGCACGTTTACTTTGAAAAAAT 180

181 TAGAGTGCTCAAAGCAGGCGTGTAGCCTGAATAATGGTGCATGGAATAATGGAATAGGAC 240

241 TTCGTTTCTATTTTGTTGGTTTTCGGAATACGAGGTAATGATTAAGAGGGACAGACGGGG 300

301 GCATTCGTATTGCGACGCTAGAGGTGAAATTCTTGGACCGTCGCAAGACGAACTACTGCG 360

361 AAAGCATTTGCCAAGAATGTTTTCATTAATCAAGAACGAAAGTTAGAGGTTCGAAGGCGA 420

421 TCAGATACCGCCCTAGTTCTAACCATAAACGATGCCAACCAGCGATCCGCCTGAGTTCCT 480

481 CAAATGACTCGGCGGGCAGCTTCCGGGAAACCAAAGTGTTTGGGTTCCGGGGGAAGTATG 540
```

```
 541 GTTGCAAAGCTGAAACTTAAAGGAATTGACGGAAGGGCACCACCAGGAGTGGAGCCTGCG 600

 601 GCTTAATTTGACTCAACACGGGAAAACTTACCCGGCCCGGACACTGGAAGGATTGACAGA 660

 661 TTAAGAGCTCTTTCTTGATTCAGTGGGTGGTGGTGCATGGCCGTTCATAGTTGGTGGAGC 720

 721 GATTTGTCTGGTTTATTCCGATAACGAACGAGACTCTAGCCTACTAAATAGGCGTCCCGA 780

 781 TCACTTCTGTCGGGCGTTCTTCTTAGAGGGACAAATGGCGTTTAGCCGCACGAGAGTGTA 840

 841 CATCACATCGTACAGCTCCCTCAACCCAGGATTCGAATTCGATCATCCGTCTCTTCGCTC 900

 901 TCTTCTCCAGAACCCAGAGGGTTTAGGCCAAATTCGGTGAATCatggcggatgacgcgaa 960
   1                                            M  A  D  D  A  K  6
 961 acagaaggctcttgatgagaaagagcgtaagaaagccgaggtcagagcacggctggaagc 1020
   7  Q  K  A  L  D  E  K  E  R  K  K  A  E  V  R  A  R  L  E  A  26
1021 cgctgctgctggcaagaaggcgaagaagggtttcatgacccccgcccgtaagaagaagtt 1080
  27  A  A  A  G  K  K  A  K  K  G  F  M  T  P  A  R  K  K  K  L  46
1081 gaggaccttgctgaggaaaaaggccgctgaagaattgaagcgagaacaagagaggaaagc 1140
  47  R  T  L  L  R  K  K  A  A  E  E  L  K  R  E  Q  E  R  K  A  66
1141 agaagagaggaagaaaactattgcaagtaggtgcggacctcagaagaatctcgatggaat 1200
  67  E  E  R  K  K  T  I  A  S  R  C  G  P  Q  K  N  L  D  G  I  86
1201 taatgaagctgaattaataaatatctgcaaagaataccatgatcgcatagctgaactgga 1260
  87  N  E  A  E  L  I  N  I  C  K  E  Y  H  D  R  I  A  E  L  E  106
1261 aggtcagaagtacgacatggaattccaggctagacacaaagaatacaagatcaatgagct 1320
 107  G  Q  K  Y  D  M  E  F  Q  A  R  H  K  E  Y  K  I  N  E  L  126
1321 gaacatccaagtcagtgatctccggggaaaattcgtcaaacctgtactgaagaaggtatc 1380
 127  N  I  Q  V  S  D  L  R  G  K  F  V  K  P  V  L  K  K  V  S  146
1381 cagattcgaatacggcaaatttgagaaactcatgcgagcagccaagaaggccgacaatga 1440
 147  R  F  E  Y  G  K  F  E  K  L  M  R  A  A  K  K  A  D  N  D  166
1441 tttccgtaccaacctgaagtccgttggaccatccacaaaatacaagcttgaggatgatgt 1500
 167  F  R  T  N  L  K  S  V  G  P  S  T  K  Y  K  L  E  D  D  V  186
1501 taaagagtctaaacccgaatgggccgcgggagtcaaggaagcagccaagaagggagaaga 1560
 187  K  E  S  K  P  E  W  A  A  G  V  K  E  A  A  K  K  G  E  E  206
1561 aTAAAAAGATTTGTGCCATTGTCTTCATATCTGTGTATATCGTCATATAAATATTCTTAC 1620

1621 ATCGGAAGAATTCTAGGTTTCTCCGACAATAAATTCAGTGGCTGAGCTTCGACGTCATCA 1680

1681 AAACCGAAGAAGCATTACTTCCAAAGATTCTACATCGAAATTCAGGAGAGAAGGAAACTT 1740

1741 GATTTCGCTGCCGGCAAGAATAGTCACGAACTTACAAATAATAGTAATCAGTACATTCAA 1800

1801 TTCCTGTAAGCTTTTAAGGCGAAAAGCTACAAATAGTTACGAAGAGCTACTAGCTAAATG 1860

1861 GAAACTTCAGCATCTATGTATTCTGTCAATATTTGGCATTGATGTATTACTACTTCCGTT 1920

1921 TATGAAGCTGACGTTTCCGTTTTAATGTTCGTTGTTAC 1958
```

Supplementary Figure 6 - Nucleotide and predicted amino acid sequences of TNNI1_As.

```
   1 CGGGCTGCAGGATTCGAATTCGTAAGAAGGAAAGGGCTCTGATTTCCTAAGTTTTCTGAA 60

  61 GAAatggctgatcaatcggaggatgaaagaagaaggcaagaagaaaaggagagaaaaaaa 120
   1    M  A  D  Q  S  E  D  E  R  R  R  Q  E  E  K  E  R  K  K  19
 121 gcagaagtgcgcaggcgcctcgaagaaaccaccaaatttaagaaatgtaaaaaaggaggt 180
  20 A  E  V  R  R  R  L  E  E  T  T  K  F  K  K  C  K  K  G  G  39
 181 ttcatgactccgcagcgaaagaagaaactccggactttactgagaaaaaaggcagcagag 240
  40 F  M  T  P  Q  R  K  K  K  L  R  T  L  L  R  K  K  A  A  E  59
 241 gagctgaaaagagaacaagagcgaaaagcagaagaaaggaagaaaatgatcactgagcgt 300
  60 E  L  K  R  E  Q  E  R  K  A  E  E  R  K  K  M  I  T  E  R  79
 301 tgtggccaacccaaaagccttgacaatgccaatgaagcaacgttacaggccatatgcaaa 360
  80 C  G  Q  P  K  S  L  D  N  A  N  E  A  T  L  Q  A  I  C  K  99
 361 gaatattacaaacgaattgctcagctcgaagatgacaagtatgacttggaatatgacgtc 420
 100 E  Y  Y  K  R  I  A  Q  L  E  D  D  K  Y  D  L  E  Y  D  V  119
 421 cgacaaaaagactttttgatcaatgaactgactatacaagtgaatgacctgaggggaaaa 480
 120 R  Q  K  D  F  L  I  N  E  L  T  I  Q  V  N  D  L  R  G  K  139
 481 tttgtcaagccaactttgaaaaaggtgtctaaatacgaaggaaaatttgagaaactaaag 540
 140 F  V  K  P  T  L  K  K  V  S  K  Y  E  G  K  F  E  K  L  K  159
```

```
541 atggttgccaaaacgacagaagtcgacttcagaacgaatctgaaatcggtcaagtcaaac 600
160 M  V  A  K  T  T  E  V  D  F  R  T  N  L  K  S  V  K  S  N   179
601 aaattcaagcttggagaagatgaagagggcaagaaacaagctcctgagtgggccaacaaa 660
180 K  F  K  L  G  E  D  E  E  G  K  K  Q  A  P  E  W  A  N  K   199
661 TGAGCCGGATAACAAGCTACCTTTTAAGAGGCAAAGGAACTGCTTTCCCAGAAACATCAG 720

721 GTGTTTAATTTAAATGCAAAGGAAGTGAGAGGACAAAATACACTGCTTAGTACTTCTCTA 780

781 TATCTTTGCCGTCCAAGTTATAAAATGTCCAGATGTGTGCCGCTGATTGCATCTCTGGTA 840

841 AAAAAAAAAAAAAACAATAAATTTATTTGAAAAGGC 876
```

Supplementary Figure 7 - Nucleotide and predicted amino acid sequences of TNNI2_As.

```
   1 TTTTTGGTAAACAGTCAGAAATTTAGGGCCGAGTTCATAATCTTAGGTGAAAATAATATA 60

  61 AAATAGCGGTTTTAGAAGAGGTATATATTTCTAATTTACTAATAAAATCATTATGAGAAT 120

 121 TGATAAATGGATTTCTGTAATAGAATTATTTAATTTTTGCTTGTTGTTGTAAAACTTCTG 180

 181 GGACACTGTTAATCCTATTTGTTTTAAAATTTTTTAGGTGAAAATGTATTTATAAATTTA 240

 241 TATTTAAAAGTTGAGTAGTACTGAATACATTCCAGAAAAACCAGATATAAAAAAAAGCGA 300

 301 AAGGAGTTTCACCTCTAAAAGAATATTAAGGAAATATTGTGAAACATAAGATTTGTATTC 360

 361 TTTTGGAAGAGGAGGAAGCCatgtggcaggagtacatcgatcaatggaggaaacagcggg 420
  1                     M  W  Q  E  Y  I  D  Q  W  R  K  Q  R  A 14
 421 ccaaggaagaggaggagcttaggaagctgaaagaacgtcaagcgagacgaaaggtaacta 480
  15   K  E  E  E  E  L  R  K  L  K  E  R  Q  A  R  R  K  V  T  R 34
 481 gagctgaacaagagaaacgtctcatggagttaaaaaggaaacaagaggaacagagagtga 540
  35   A  E  Q  E  K  R  L  M  E  L  K  R  K  Q  E  E  Q  R  V  R 54
 541 gagaaatcgaagagaaaaagcaaaaagaagcggaagcgaaaaggaaacgtctggaagaag 600
  55   E  I  E  E  K  K  Q  K  E  A  E  A  K  R  K  R  L  E  E  A 74
 601 cagaaaggaagaggcaggcaatgcttgaggagcaaaggaaacagaaggaaggtgtgaaac 660
  75   E  R  K  R  Q  A  M  L  E  E  Q  R  K  Q  K  E  G  V  K  P 94
 661 ccaacttcgtcatccagaagaaagctgaaggtggtgcccctgttgtatctcatcatccag 720
  95   N  F  V  I  Q  K  K  A  E  G  G  A  P  V  V  S  H  H  P  G 114
 721 gcggatttgacaagctaagcactttggaacaagccaggaacgagctgctcaaatccaagg 780
 115   G  F  D  K  L  S  T  L  E  Q  A  R  N  E  L  L  K  S  K  E 134
 781 agcaactggcagaagacaaagccattgctctcacttacagagtgaaacctctgaacattg 840
 135   Q  L  A  E  D  K  A  I  A  L  T  Y  R  V  K  P  L  N  I  E 154
 841 aaggccttggttccggaaaactcaaggatgttggcgaggaattgtggaacaagattgttc 900
 155   G  L  G  S  G  K  L  K  D  V  G  E  E  L  W  N  K  I  V  Q 174
 901 agcttgagagcgagaaatatgacttggaggaaaagatgaagagacaggactacgatctta 960
 175   L  E  S  E  K  Y  D  L  E  E  K  M  K  R  Q  D  Y  D  L  R 194
 961 gggagttgactgagagacagaagcaaattaataggcagaaggcactcaagaaaggcattg 1020
 195   E  L  T  E  R  Q  K  Q  I  N  R  Q  K  A  L  K  K  G  I  D 214
1021 atccagcagaagctgagggaaaatacccgcctaaaatccacgtggctagcaagtttgagc 1080
 215   P  A  E  A  E  G  K  Y  P  P  K  I  H  V  A  S  K  F  E  R 234
1081 gacgggttgacaggagaacattcggcgacaagaaacagttttatgatggaggtcttgagg 1140
 235   R  V  D  R  R  T  F  G  D  K  K  Q  F  Y  D  G  G  L  E  E 254
1141 aggacattaaagctaaattggagaaatgctggaaagacagaatgacctcgtttaaggaaa 1200
 255   D  I  K  A  K  L  E  K  C  W  K  D  R  M  T  S  F  K  E  R 274
1201 gaggtcctaaacagctacccaaatgggatcccactgctccgaaagtcaaagaagttattg 1260
 275   G  P  K  Q  L  P  K  W  D  P  T  A  P  K  V  K  E  V  I  E 294
1261 aggcaaggacgtacgatgaagatgatgaccttcttgacttagaaccaccatccttcggag 1320
 295   A  R  T  Y  D  E  D  D  D  L  L  D  L  E  P  P  S  F  G  A 314
1321 caccagcagagcccgagcccgctcccagagctccttcacctcctccacctcaggaggaag 1380
 315   P  A  E  P  E  P  A  P  R  A  P  S  P  P  P  P  Q  E  E  E 334
1381 aagaagaagaagaagaggaagaggaggaggatgaagaggaagaagaggaagaaTAAACGA 1440
 335   E  E  E  E  E  E  E  E  E  D  E  E  E  E  E  E  E         351
1441 CTAATTTTTGGAAAAAGGATCTGAATTTTGCGGGTTTCGAGTGTAGAACTGAGTAGTCGT 1500

1501 GTCTTCCTCATATTTCTTTAAAAAAAAACGGCAAACAAAAAAAAAAAAAAATCACTGCAG 1560

1561 CTGTCATTATGTCTTCTTCTTGTTGAGACATCGTCACATGCACCAGAATAAGTCGGCCTG 1620
```

```
1621 GAAAACTGCTGAAGAAAATATTAATACAGGGAATACCCTGTAAAGGGGGAAAAGAAGTTT 1680

1681 CTATGTCTTTTTCGATTCATTTACCTGCGGCTTCTGTGTCTGAAAATCATATCATCATAA 1740

1741 AATTTTTCTGGAAAATTTAAATAACATTACCGATGGGCAATCAATTTTCTTCGAAGATTT 1800

1801 GTAAAATTTTTTCTGCTGAAAATGCATTCGGAAAACTACTGGTTGACGGAATTTTGTAAC 1860

1861 GTATTTCAGAAAAAATGAAATGTACATGGAATGCTATGTATATAGGATCGTTTAGATCTT 1920

1921 GTAAGCATGGTGTTGCCTGTTAATGTAATCCCATATTTATTAAAACTGCATTGTTTTCTT 1980

1981 TCAAAAAAAAAAAAAAAAAAACTGAGAATTCGAATTCCTGCAGCCCGGGGGATCCA 2035
```

Supplementary Figure 8 - Nucleotide and predicted amino acid sequences of TNNT1_As.

```
   1 CAAGAAGTTTATTTTCAATGCTAAGAAGTGAACAATTCTGAGTGACATCTTACATACTGA 60

  61 AACATTTTCCTGTAAACTGTAGAACATTTACCTGATTTACACATTTGAAAAGCAATACCT 120

 121 ATAAGGACCCTTCTTGTTATAATATACAACAAAATTAACTAGTATTTTAAATGATAAAAA 180

 181 TCCAAATGTAAGCATAATTCTGCAATGTTAAGTGAAAAAAAAAATAAAATTGCAATGATA 240

 241 TAGACACCATACAAAAGTTAATACTGTGGCATATTCTCTTCTTGAGAATGATAACCAAGT 300

 301 TTCAAACACTGAGAAGAATGGCAATATAATAAATTTTTCTAAAAGGAAAAAAAGTTGTAA 360

 361 ATATATGTTTAAGTATAACTTTCAAGGTGATCCGCGGAAGCAACTCGCACTCGCTCTCTC 420

 421 TCCCGGATCTTGAGCTGGTCGCTGCTCTCGTGTCTTCCTCTTTCATCTCTGCTCTCGGTG 480

 481 TTCACCCGTCCGACGATCCACGatggaggccatcaagaagaagatgcaaggtatgaagtt 540
   1                       M  E  A  I  K  K  K  M  Q  G  M  K  L  13
 541 ggagaaggacaatgccgtcgacagggcagaaaccgcagagaaccagtcacgagatgcaaa 600
  14  E  K  D  N  A  V  D  R  A  E  T  A  E  N  Q  S  R  D  A  N  33
 601 tctacgagccgacaaggctgaagaggaagtacgttcgctgcaaaagaagatccagcaaat 660
  34  L  R  A  D  K  A  E  E  E  V  R  S  L  Q  K  K  I  Q  Q  I  53
 661 tgaaaatgagttggatcaagtgcaggagcagctggctcaagccaacaacaaactggaaga 720
  54  E  N  E  L  D  Q  V  Q  E  Q  L  A  Q  A  N  N  K  L  E  E  73
 721 gaaggacaaagctcttcaagccgctgaaggtgaagtagctgctctgaacaggcgcatcca 780
  74  K  D  K  A  L  Q  A  A  E  G  E  V  A  A  L  N  R  R  I  Q  93
 781 actgctagaggaggaccttgagcgttcagaagagcgtctcaagactgcaacagcaaaact 840
  94  L  L  E  E  D  L  E  R  S  E  E  R  L  K  T  A  T  A  K  L  113
 841 tgaagaagcctcccaagctgcagacgaaagtgaacgtatgcgtaagatgcttgagcacag 900
 114  E  E  A  S  Q  A  A  D  E  S  E  R  M  R  K  M  L  E  H  R  133
 901 gagtataacagatgaggaacgtatggatgctcttgaagaccagcttaaagaggctaggtt 960
 134  S  I  T  D  E  E  R  M  D  A  L  E  D  Q  L  K  E  A  R  L  153
 961 gatggctgaggaggctgatcgcaaatatgatgaggttgcccgtaagatggcaatggttga 1020
 154  M  A  E  E  A  D  R  K  Y  D  E  V  A  R  K  M  A  M  V  E  173
1021 agctgatttggaacgtgctgaagagcgtgcagagacaggtgaaaacaaaatcgtggagct 1080
 174  A  D  L  E  R  A  E  E  R  A  E  T  G  E  N  K  I  V  E  L  193
1081 tgaggaagaactgagagttgttggcaacaacttaaagtctctggaagtcagtgaagagaa 1140
 194  E  E  E  L  R  V  V  G  N  N  L  K  S  L  E  V  S  E  E  K  213
1141 ggccttacaaaaggaagaaacctatgaaatgaccatcaggcagatgacccagaggctcca 1200
 214  A  L  Q  K  E  E  T  Y  E  M  T  I  R  Q  M  T  Q  R  L  Q  233
1201 ggaggctgaagccagagcagaatttgctgagaggtctgtacaaaaactccagaaggaggt 1260
 234  E  A  E  A  R  A  E  F  A  E  R  S  V  Q  K  L  Q  K  E  V  253
1261 tgacagattggaagatgaactggttcaagaaaaggagaaatacaaggccatttccgacga 1320
 254  D  R  L  E  D  E  L  V  Q  E  K  E  K  Y  K  A  I  S  D  E  273
1321 attggaccagacttttgcagagcttactggctatTAAAAGCTTTCCATGCTTATGTATTT 1380
 274  L  D  Q  T  F  A  E  L  T  G  Y                             284
1381 CTGTGTATCTTGACAAATTCCATTTTGTATGTCACTTACAACATGGATATGCTATCAACC 1440

1441 ATCCAAATGTGTTTTTGAAAGTGGTATTAGTGGCTAATTGGCATCCTGTTTCTTTTAGTT 1500

1501 TTCATAAACAGATTTGGTTCAGTATGGTTTGTATAGGCATGCCTTGATTTGTTCTGCAAC 1560

1561 AGATCTGTGAAATTGGAGTTGTAGAATTCTGACTACAGGAAATGTCCAGATGAAGTTTAA 1620
```

1621 TTAATGAAAAAGAAACCTAAGTTATCTGAAATTGTAAATTTGAAATATTCATTATAAAAG 1680

1681 ACAACATTAAAATATATTCATTGTAAAAAAA 1711

Supplementary Figure 9 - Nucleotide and predicted amino acid sequences of TPM2_As.
