# Supplementary material for: Analysis of tarantula skeletal muscle protein sequences and identification of transcriptional isoforms
Source: BMC Genomics. 2009 Mar 19;10:117. doi: 10.1186/1471-2164-10-117 (PMC2674065; doi:10.1186/1471-2164-10-117)
Supplement: Additional file 4 — Supplementary figures (sequence alignments). The alignments of tarantula myofibrillar proteins with other species. [file 1471-2164-10-117-S4.pdf]

```
                        *        20         *        40         *
MLE_AEQIR       MPKLSQDEIDDLKDVFELFDFWDGRDGAVDAFKLGDVCRCLGINPRNEDV   50
MLE1_As         .AD.KAA..EKA.EH.....-.E.-E.K...RD....L.S.DCK.TLAM.   48
Brazil.RBC.ELC  .AD.KAA..EKA.EH.....-.E.-E.K...RD....L.S.DCK.TLAM.   48
As.Contig98     .AD.KSY..EEC.AA.....-TT.-E.K...VY.TP.L.A.D.CVT.DTA   48
Q3L6K7_9ACAR    .AD.KP...EQA.EH.....-MCA-E.K..CAD..S.L.S.D.R.TKAL.   48
MLC1_DROME      .AD.PKR..E..EF....MG-SP.-E.-...VD...AL.A.N...TLAL.   47

                       60         *        80         *       100
MLE_AEQIR       FAVGGTHKMGEKSLPFEEFLPAYEGL-MDCEQGTFADYMEAFKTFDREGQ   99
MLE1_As         KKN...D.R...K.TL.....I.SQ.KKEK.V..LE....GL.V...AEN   98
Brazil.RBC.ELC  KKN...E.K...K.TL.....I.SQ.KKEK.I..L-....GL.V...AEN   97
As.Contig98     EKA.AAK.E...M.T.D....I.SDCRKSKDM.QIE.L.              87
MLE2_As                                       DM.QIE.L.-GL.V...NEN   19
Q3L6K7_9ACAR    EKN..CA.K...K.TL.....I.SQ.KK.KD...H.....GL.V...AEN   98
MLC1_DROME      EK....K.RN..K.KLD....I.SQ.KKEK...C.E....CL.L....EN   97

                        *       120         *       140         *
MLE_AEQIR       GFISGAELRHVLTALGERLSDEDVDEIIKLTDLQEDLEGNVKYEDFVKKV  149
MLE1_As         .T.LA...A...LS.......IECE.....C.--..DD.F....P...T.  146
Brazil.RBC.ELC  .T.LA...A...LS.......IECE.....C.--..DD.F....PM..T.  145
MLE2_As         .T.MA...A...LS.....K.PE....FTACCPP..ED.Y....AL..N.   69
Q3L6K7_9ACAR    .Q.ME...A...LS.......AE.....HDCAG.V.ED.F....M...N.  148
MLC1_DROME      .T.LL...Q.A.L....S.D..Q.ET.FADCMDP..D..F.P.SQ..Q..  147

                      160
MLE_AEQIR       MAGPYPDK      157
MLE1_As         .......EGK    156
Brazil.RBC.ELC  ......EESK    155
MLE2_As         F.....SEEPPK   81
Q3L6K7_9ACAR    ......EEQKDK  160
MLC1_DROME      .SD.VVFD      155
```

Supplementary Figure 1 -Alignment of tarantula myosin essential light chains with other myosin light chains.

The abbreviation and Swiss-Prot accession number for the ELC sequences aligned are: MLE_AEQIR, *Aequipecten irradians*(Bay scallop) striated adductor muscle ELC (P07291); MLE1_As, MLE2_As, As.Contig98, *Aphonopelma sp.* (Tarantula) skeletal muscle ELCs; Brazil.RBC.ELC: the minor ELC found in hemocytes of *Acanthoscurria gomesiana* (tarantula) based on EST analysis; Q3L6K7_9ACAR, *Haemaphysalis* qinghaiensis(tick) myosin ELC (Q3L6K7); MLC1_DROME, *Drosophila melanogaster* (Fruit fly) myosin ELC (P06742). Dot indicates identity with the top sequence; Dash indicates a gap inserted for spacing purposes. The calcium-binding residues conserved in scallops are in bold.

```
                       *        20         *        40         *
MYS_AEQIR         MNIDFSDPDFQYLAVDRKKLMKEQTAA-FDGKKNCWVPDEKEGFA     44
MYSA_DROME   MPKPVA.QEDE..T-P..F.SLEQRRID.SKP-Y.S..S..I......YL     48
MYHHead4_As      MAIE.DP..T-EF.F.SLEQKR.D..KP-Y....MV......D..V     44
MYSS_CHICK   ASPDAEMAA.GEAA-P..RKSE.ERIEA.NKP-..A.SSVF.VHP..S.V     48
MYHB_CHICK        SQKPL..DE-KF.F..KNFVNNPLAQ.DWSA..LV...S..H..E     44

                     60         *        80         *       100
MYS_AEQIR    SAEIQSSKGDEITVKIVADSSTRTVKKDDIQSMNPPKFEKLEDMANMTYL     94
MYSA_DROME   LG..KAT...IVS.GL-QGGEV.DI.SEKVEKV.......I....D..V.     97
MYHHead4_As  LGN.V.T...MA..DL-PGGE-KVM..ELL.QV.......CD.VSSL...     92
MYSS_CHICK   KGT...KE.GKV...T-EGGE.L...E.QVF......YD.I....M..H.     97
MYHB_CHICK   A.S.KEE....V..ELQENGKKV.LS.....K......S.V....EL.C.     94

                                   ATP binding site I
                       *       120         *       140         *
MYS_AEQIR    NEASVLYNLRSRYT-SGLIYTYSGLFCIAVNPYRRLPIYTDSVIAKYRGK    143
MYSA_DROME   .TPC..H...Q..Y-AK..........V.I...K.Y.V..NRCAKM....    146
MYHHead4_As  ......H..KD..INCN..........V.I...K.F....NR.VQM.K..    142
MYHHead1_As                              .I...K.F....NRTVQI.K.R     22
MYSS_CHICK   H.PA.....KE..A-AWM.........VT....KW..V.NPE.VLA....    146
MYHB_CHICK   ......H...E..F-............VVI...KQ....SEKI.DM.K..    143

                                    ATP binding site II
                    160         *       180         *       200
MYS_AEQIR    RKTEIPPHLFSVADNAYQNMVTDRENQSCLITGESGAGKTENTKKVIMYL    193
MYSA_DROME   .RN.V...I.AIS.G..VD.L.NHV...M..................A.F    196
MYHHead4_As  .RG.M...I.A.S.G..ND.LQN.....M..................A.F    192
MYHHead1_As  .R..V.....AIS.G..SA.LAN.....M..................A.Y     72
MYSS_CHICK   KRQ.A...I..IS.....F.L.......I...........V...R..Q.F    196
MYHB_CHICK   KRH.M...IYAI..T..RS.LQ...D..I.C................Q..    193

                    Loop I                         ATP binding
                       *       220         *       240         *
MYS_AEQIR    AKVAC-AVKKKDEEASDKKE---GSLEDQIIQANPVLEAYGNAKTTRNNN    239
MYSA_DROME   .T.GA----S.KTDEAA.SK---......VV.T......F.....V..D.    239
MYHHead4_As  .N.GAVTKSS..KKDAAS.K---.N....V..T......F.....V..D.    239
MYHHead1_As  .N.GAATP.PGK.APTKE.K---AT....VV.T......F.....V..D.    119
MYSS_CHICK   .TI.A-SGE..K..Q.G.MQ---.T......S...L...F.....V..D.    242
MYHB_CHICK   .V..SSHKG...TSITQGPSFSY.E..K.LL....I...F.....VK.D.    243


             site III
                    260         *       280         *       300
MYS_AEQIR    SSRFGKFIRIHFGPTGKIAGADIETYLLEKSRVTYQQSAERNYHIFYQIC    289
MYSA_DROME   .................L............A..IS...L..S.......M    289
MYHHead4_As  ..............M..L...........                         268
MYHHead2_As                                               .IKL.      5
MYHHead1_As  ..............M..L............A..IS..TL..S......LM    169
MYSS_CHICK   .............A...L.S..............F.LP...S.......M    292
MYHB_CHICK   ..........N.DV..Y.V..N..........AIR.AKD..TF....YLI    293
```

```
                       *       320         *       340         *
MYS_AEQIR    SNAIPELNDVMLVTPDSGLYSFINQGCLTVDNIDDVEEFKLCDEAFDILG  339
MYSA_DROME   .GSV.GVK.IC.L.DNIYD.HIVS..KV..AS...A...S.T.Q......  339
MYHHead2_As  LGK.SN.KEKV.LSNNIND.H.VS..KTAIPGV..G.DMLVT.NG..V..   55
MYHHead1_As  .GG.EN.KADL.LSD.IYD.H.VS..KIEIPGV.NA..LR.T.T......  219
MYSS_CHICK   ..KK...I.ML.I.TNPYD.HYVS..EI..PS...Q..LMAT.S.I....  342
MYHB_CHICK   AG.SEQMRNDL.L-EGFNN.T.LSN.HVPIPAQQ.D.M.QETL..MT.M.  342

                    360         *       380         *       400
MYS_AEQIR    FTKEEKQSMFKCTASILHMGEMKFKQRPREEQAESDGTAEAEKVAFLCGI  389
MYSA_DROME   ...Q..EDVYRI..AVM...G......G......Q..EE.GGR.SK.F.C  389
MYHHead2_As  ..D...LNIY.I.G.VM.L..L............A...EPG....H.L.L  105
MYHHead3_As                                   .A..KRKVRELPTWL.V   17
MYHHead1_As  .SH.Y.TDVY.I...CM.L...............A...E.G.R..H.L.V  269
MYSS_CHICK   .SAD..TAIY.L.GAVM.Y.NL....KQ......P...EV.D.A.Y.M.L  392
MYHB_CHICK   ..E..QT.ILRVVS.V.QL.NIV..KERNTD..SMPDNTA.Q..CH.M..  392

                      Actin binding site I
                      *       420         *       440         *
MYS_AEQIR    NAGDLLKALLKPKVKVGTEMVTKGQNMNQVVNSVGALAKSLYDRMFNWLV  439
MYSA_DROME   DTAE.Y.N....RI...N.F..Q.R.VQ..T..I...C.GVF..L.K...  439
MYHHead2_As  ..A..Y.N.....I...N.L..Q.R.KE...Y.....S.AM...L.K...  155
MYHHead3_As  ..A..Y.N.V...I...N....Q.R.AT..SY...G...AMF..T.K...   67
MYHHead1_As  ..A..Y.N.V...I...N....Q.R.AT..SY...G...AMF..T.M...  319
MYSS_CHICK   .SAE.....CY.R....N.F.....TVS..H........AV.EK..L.M.  442
MYHB_CHICK   .VT.FTRSI.T.RI...RDV.Q.A.TKE.ADFAIE....AKFE.L.R.IL  442

                    460         *       480         *       500
MYS_AEQIR    RRVNKTLD-TKAKRNYYIGVLDIAGFEIFDFNSFEQLCINYTNERLQQFF  488
MYSA_DROME   KKC.E...-.QQ..QHF............EY.G.......F...K.....  488
MYHHead2_As  K...E...-..Q..Q.F.......................F...K.....  204
MYHHead3_As  K.L.E...-..Q..Q.F.............Y.G.......F...K.....  116
MYHHead1_As  K.L.E...-..Q..Q.F.............Y.G.......F...K.....  368
MYSS_CHICK   I.I.QQ..-...QP.Q.F......................F...K.....  491
MYHB_CHICK   T....A..K..RQGASFL.I.........EI.............K...L.  492

                      *       520         *       540         *
MYS_AEQIR    NHHMFILEQEEYKKEGIAWEFIDFGMDLQMCIDLIEKPM---GILSILEE  535
MYSA_DROME   ..I..VM..........N.D........LA.........---........  535
MYHHead2_As  .....V.......R...E.T.....L...A.........---.V......  251
MYHHead3_As  .....V.......R...D.V.....L..AA..E......---........  163
MYHHead1_As  .....V.......R...D.V.....L..AA..G......---........  415
MYSS_CHICK   .....V...........E..........AA..E......---..F.....  538
MYHB_CHICK   ..T.........QR...E.N.....L...P..E...R.TNPP.V.AL.D.  542

                           Actin binding site II and III
                    560         *       580         *       600
MYS_AEQIR    ECMFPKADDKSFQDKLYQNHMGKNRMFTKPGKPTRPNQGPAHFELHHYAG  585
MYSA_DROME   .S.....T.QT.SE..TNT.L..SAP.Q..-..PK.G.QA...AIA....  584
MYHHead2_As  .S.....T..T.EE..KT..L..SPN.V..-..PK.G.QE...AIV....  300
MYHHead3_As  .S.....T........NA..L..SPN.V..-..PK.G.QE...SIA....  212
MYHHead1_As  .S.....T........NA..L..SPN.V..-..PK.G.QE...SIA....  464
MYSS_CHICK   .......T.T..KN...DQ.L..SNN.Q..-..AK-GKAE...S.V....  586
MYHB_CHICK   ..W....T.T..VE..I.EQ-.NHAK.Q.S-.QLKDKTE---.CIL....  587
```

```
                                                       Loop II
                      *        620        *        640        *
MYS_AEQIR    NVPYSITGWLEKNKDPINENVVALLGASKEPLVAELFKAPE--------E  627
MYSA_DROME   C.S.N...........L.DT..DQFKK.QNK.LI.I.ADHA---G----Q  627
MYHHead2_As  T...NLN...----------------------------------------  310
MYHHead3_As  T...N...........V.DT..DQFKKGSNK..Q.I.EDHP---GLGAE.  259
MYHHead1_As  T...N...........V.DT..DQFKKGSNK..Q.I.EDHP---GLGAE.  511
MYSS_CHICK   T.D.N.S.........L..T.IG.YQK.SVKTL.L..ATYG---G----.  629
MYHB_CHICK   K.T.NASA..T..M..L.D..TS..NQ.SDKF..D.W.DVDRIVGLDQMA  637

                    660         *       680         *       700
MYS_AEQIR    PAGGGKKKKG---KSSAFQTISAVHRESLNKLMKNLYSTHPHFVRCIIPN  674
MYSA_DROME   SG..EQA.G.RGK.GGG.A.V.SAYK.Q..S..TT.R..Q..........  677
MYHHead3_As  KG....GGG.R-K.GAS...V..LY..Q..R..AS.H..Q..........  308
MYHHead1_As  KG....GGG.R-K.GAS...V..LY..Q.....TT.R..A..........  560
MYSS_CHICK   AE...G..G.K-K.G.S...V..LF..N.....A..R.............  678
MYHB_CHICK   KMTESSLPSASKT.KGM.R.VGQLYK.Q.T...TT.RN.N.N........  687

                            SH2       SH1
                      *        720        *        740        *
MYS_AEQIR    ELKQPGLVDAELVLHQLQCNGVLEGIRICRKGFPSRLIYSEFKQRYSILA  724
MYSA_DROME   .M....V...H..M...T................N.MM.PD..M..Q..N  727
MYHHead3_As  ......VI.SG..M...T................N.MV.PD.....T...  358
MYHHead1_As  .T.S..VI.SG..M...T................N.MV.PD.....T...  610
MYSS_CHICK   .T.T..AMEH.......R..................VL.AD.....RV.N  728
MYHB_CHICK   HE.RA.KL..H...E..R.............Q...N.IVFQ..R...E...  737

                    760         *       780         *       800
MYS_AEQIR    PNAIPQG-FVDGKTVSEKILAGLQMDPAEYRLGTTKVFFKAGVLGNLEEM  773
MYSA_DROME   .RG.-KD-LDCP.KA.KVLIESTELNEDL....H.....R.....QM..F  775
MYHHead3_As  AS.V.K.-...A.K.T.AV.GAI.L.AND....N.....R.....R....  407
MYHHead1_As  AS.V.K.-...                                         620
MYSS_CHICK   AS...E.Q.M.S.KA...L.GSIDV.HTQ..F.H........L..L....  778
MYHB_CHICK   A....K.-.M...QACILMIKA.EL..NL..I.QS.I..RT...AH...E  786

                            ELC binding site          RLC binding
                      *        820        *        840        *
MYS_AEQIR    RDERLSKIISMFQAHIRGYLIRKAYKKLQDQRIGLSVIQRNIRKWLVLRN  823
MYSA_DROME   .....G..M.WM..WA....S..GF....E..VA.K.V...L..Y.Q..T  825
MYHHead3_As  ..D..G..VTWL..W..W..SK.EF....E..VA.L.....L..F.T...  457
MYSS_CHICK   ..DK.AE..TRT..RC..F.M.VE.RRMVER.ESIFC..Y.V.SFMNVKH  828
MYHB_CHICK   ..LKITDV.IA...QC....A...FA.R.Q.LTAMK.....CAAY.K...  836

              site
                    860
MYS_AEQIR    WQWWKLYSKVK  834
MYSA_DROME   .P.Y..WQ...  836
MYHHead3_As  .L.Y...I...  468
MYSS_CHICK   .P.M..FF.I.  839
MYHB_CHICK   ....R.FT...  847
```

Supplementary Figure 2 - Alignment of tarantula myosin heavy chain S1 fragments with known myosin heavy chains.

The abbreviation and Swiss-Prot accession number for the myosin heavy chain sequences aligned are: MYS_AEQIR, *Aequipecten irradians* (Bay scallop) myosin heavy chain, striated muscle (P24733); MYSA_DROME, *Drosophila melanogaster* (Fruit fly) myosin heavy chain, muscle (P05661); MYHHead1_As, MYHHead2_As, MYHHead3_As, MYHHead4_As, *Aphonopelma sp.* (Tarantula) skeletal muscle myosin heavy chain isoforms; MYSS_CHICK, *Gallus gallus* (Chicken) myosin heavy chain, skeletal muscle, adult (P13538); MYHB_CHICK, *Gallus gallus* (Chicken) myosin heavy chain, gizzard smooth muscle (P10587). Dot indicates identity with the top sequence; Dash indicates a gap inserted for spacing purposes.The ATP and actin binding regions, the loop regions, the light chain binding regions are underlined.

```
                         *        20         *        40         *
MYS_AEQIR       PLLSIARQEEEMKEQLKQMDKMKEDLAKTERIKKELEEQNVTLLEQKNDL   884
MYSA_DROME      ...NVS.I.D.IARLEEKAK.AE.LH.AEVKVR....AL.AK..AE.TA.   886
MYHhead3_S2_As  ...TM.KV.D.L.ALEEKLK.AL.S.E.E.KVR.DI.V...K..QE....    50
MYSS_CHICK      ...KS.ES.K..ANMKEEFE.T..E...S.AKR.....KM.V..QE....   898
MYHB_CHICK      ...QVT......QAKDEELQRT..RQQ.A.AEL....QKHTQ.C.E..L.   897

                        60         *        80         *       100
MYS_AEQIR       FLQLQTLEDSMGDQEERVEKLIMQKADFESQIKELEERLLDEEDAAADLE   934
MYSA_DROME      LDS.SGEKGALQ.YQ..NA..TA..N.L.N.LRDIQ...TQ....RNQ.F   936
MYHhead3_S2_As  ....ESERSGA..V...LT.A.SM.N.L.G.VQ..Q...SR....HSN.S   100
MYSS_CHICK      Q..V.AEA..LA.A...CDQ..KT.IQL.AK...VT..AE...EIN.E.T   948
MYHB_CHICK      QEK..AETELYAEA..MRVR.AAK.QEL.EILH.M.A.IEE..ERSQQ.Q   947

                         *       120         *       140         *
MYS_AEQIR       GIKKKMEADNANLKKDIGDLENTLQKAEQDKAHKDNQISTLQGEISQQDE   984
MYSA_DROME      QQ...ADQEISG.....E...LNV........T..H..RN.ND..AH...   986
MYHhead3_S2_As  SVR..LDGEIS....E.E..QLVI..T.....A..H..RN.ND..AH...   150
MYSS_CHICK      AK.R.L.DECSE.....D...L..A.V.KE.HATE.KVKN.TE.MAVL..   998
MYHB_CHICK      AE....QQQMLD.EEQLEEE.AAR..LQLE.VTA.GK.KKMEDD.LIMED   997

                       160         *       180         *       200
MYS_AEQIR       HIGKLNKEKKALEEANKKTSDSLQAEEDKCNHLNKLKAKLEQALDELEDN  1034
MYSA_DROME      L.N.......MQG.T.Q..GEE...A...I.....V......T......S  1036
MYHhead3_S2_As  L.N...R...QMQ.MGQ..GED...T...L..M..V......T......S   200
MYSS_CHICK      T.A..T......Q..HQQ.L.D..V....V.T.T.A.T....QV.D..GS  1048
MYHB_CHICK      QNN..T..R.L...RVSDLTTN.AE..E.AKN.T...N.H.SMIS...VR  1047

                         *       220         *       240         *
MYS_AEQIR       LEREKKVRGDVEKAKRKVEQDLKSTQENVEDLERVKRELEENVRRKEAEI  1084
MYSA_DROME      .............S.....G...L...A.A....N.K...QTIQ..DK.L  1086
MYHhead3_S2_As  ......L......T.....G...LA..A.A...KN.K...QALQ...K.M   250
MYSS_CHICK      ..Q...L.M.L.R....L.G...LAHDSIM...ND.QQ.D.KLKK.DF..  1098
MYHB_CHICK      .KK.E.S.QEL..I...L.GESSDLH.QIAE.QAQIA..KAQLAK..E.L  1097

                       260         *       280         *       300
MYS_AEQIR       SSLNSKLEDEQNLVSQLQRKIKELQARIEELEEELEAERNARAKVEKQRA  1134
MYSA_DROME      ..ITA......VV.LKH..Q..............V....Q....A.....  1136
MYHhead3_S2_As  A..SA......G..AK..KQ...................Q....A.....   300
MYSS_CHICK      .QIQ..I....A.GM...K...............I....TS...A..H..  1148
MYHB_CHICK      QAALAR....TSQKNNALK..R..ESH.SD.Q.D..S.KA..N.A...KR  1147

                         *       320         *       340         *
MYS_AEQIR       ELNRELEELGERLDEAGGATSAQIELNKKREAELLKIRRDLEEASLQHEA  1184
MYSA_DROME      D.A..........E....................S.L.......NI...S  1186
MYHhead3_S2_As  D.A..I...S...E.........V....R....MA.L......SNI...Q   350
MYSS_CHICK      D.S.....IS...E......A....M.......FQ.M.......T.....  1198
MYHB_CHICK      D.SE...A.KTE.EDTLDT.AT.Q..RA...Q.VTVLK.A...ETRT...  1197
```

```
                       360         *         380         *         400
MYS_AEQIR       QISALRKKHQDAANEMADQVDQLQKVKSKLEKDKKDLKREMDDLESQMTH  1234
MYSA_DROME      TLAN.....N..VA...E.....N.L.A.A.H.RQTCHN.LNQTRTACDQ  1236
MYHhead3_S2_As  TLAN.....N.VVA.LSE.I...N.H.AR...E.AQM.G.L..VR.SVD.   400
MYHTail1_As                                   ..E.SQM.G.L...R.SVD.    20
MYSS_CHICK      TAA......A.STA.LGE.I.N..R..Q....E.SE..M.I...A.N.ES  1248
MYHB_CHICK      .VQEM.Q..TQ.VE.LTE.LE.FKRA.AN.D.T.QT.EKDNA..ANEIRS  1247

                         *       420         *       440         *
MYS_AEQIR       NMKNKGCSEKVMKQFESQMSDLNARLEDSQRSINELQSQKSRLQAENSDL  1284
MYSA_DROME      LGRD.AAQ..IA..LQHTLNEVQSK.DETN.TL.DFDAS.KK.SI.....  1286
MYHhead3_S2_As  VN.E.ANA..QA..L.M.LTE.QGKMDEAH..LGDFDAA.K..TV..TE.   450
MYHTail1_As     VN.E.ANA..QT..M.I.LM.MQGK.DEAN..LADFDAS.K..TQ.....    70
MYSS_CHICK      VS.A.ANL..MCRTL.D.L.EIKTKE.QN..M..D.NT.RA...T.TGEY  1298
MYHB_CHICK      LSQA.QDV.HKK.KL.V.LQ..QSKYS.GE.VRT..NEKVHK..I.VENV  1297

                       460         *         480         *         500
MYS_AEQIR       TRQLEDAEHRVSVLSKEKSQLSSQLEDARRSLEEETRARSKLQNEVRNMH  1334
MYSA_DROME      L....E..SQ..Q...I.IS.TT....TK.LAD..S.E.AT.LGKF..LE  1336
MYHhead3_S2_As  ........SQ..Q...L.TS                                 470
MYHTail1_As     .....E..AQ.NQ.G.L.TS.LT..DE.K.TAD..S.E.AATMSRF..IE   120
MYSS_CHICK      S..A.EKDALI.Q..RG.QGFTQ.I.ELK.H....IK.KNA.AHALQSAR  1348
MYHB_CHICK      .SL.NE..SKNIK.T.DVAT.G...Q.TQEL.Q....QKLNVTTKL.QLE  1347

                         *       520         *       540         *
MYS_AEQIR       ADMDAIREQLEEEQESKSDVQRQLSKANNEIQQWRSKFESEGANRTEELE  1384
MYSA_DROME      H.L.NL...V...A.G.A.L........A.A.V....Y..D.VA.S....  1386
MYHTail1_As     H...TL...I.....A.A.L......S.A.V.L....Y....LA.L....   170
MYSS_CHICK      H.CELL...Y.....A.GEL..A.....S.VA...T.Y.TDAIQ......  1398
MYHB_CHICK      D.KNSLQ...D..V.A.QNLE.HI.TLTIQLSDSKK.LQEFTATVET-M.  1396

                       560         *         580         *         600
MYS_AEQIR       DQKRKLLGKLSEAEQTTEAANAKCSALEKAKSRLQQELEDMSIEVDRANA  1434
MYSA_DROME      EA....QAR.A...E.I.SL.Q..IG...T.Q..ST.V..LQL.......  1436
MYHTail1_As     EN....QA..Q..DE.I.QL.T..GS...T.T...G.V.......EK..S   220
MYSS_CHICK      EA.K..AQR.QD..EHV..V....AS...T.Q...N.V..LMVD.E.S..  1448
MYHB_CHICK      EG.K..QREIESLT.QF.EKA.SYDK...T.N......D.LVVDL.NQRQ  1446

                         *       620         *       640         *
MYS_AEQIR       SVNQMEKKQRAFDKTTAEWQAKVNSLQSELENSQKESRGYSAELYRIKAS  1484
MYSA_DROME      IA.AA....K....IIG..KL..DD.AA..DA....C.N..T..F.L.GA  1486
MYHTail1_As     LA.AL....KS...VV...KQ..DD.AA..DA..R.C.N..T.VFKLRTQ   270
MYSS_CHICK      ACAALD...KN...IL...KQ.YEET.T...A......SL.T..FKM.NA  1498
MYHB_CHICK      L.SNL....KK..QML..EKNISSKYAD.RDRAEA.A.EKETKALSLARA  1496
```

```
                         660        *       680        *       700
MYS_AEQIR       IEEYQDSIGALRRENKNLADEIHDLTDQLSEGGRSTHELDKARRRLEMEK  1534
MYSA_DROME      Y..G.EQLE.V.........VK..L..IG....NI..IE...K...A..  1536
MYHTail1_As     F..S.EHYES.K......Q...K..M...G....NV...E.S.K......   320
MYSS_CHICK      Y..SL.HLET.K......QQ..A...E.IA...KAV...E.VKKHV.Q..  1548
MYHB_CHICK      L..ALEAKEE.E.T..M.KA.ME..VSSKDDV.KNV...E.SK.T..QQV  1546

                        *       720        *       740        *
MYS_AEQIR       EELQAALEEAEGALEQEEAKVMRAQLEIATVRNEIDKRIQEKEEEFDNTR  1584
MYSA_DROME      D..........A......N..L.....LSQ..Q...R.........E...  1586
MYHTail1_As     ...........A......N..L.....LSQI.Q...R.L.D.....E...   370
MYHTail2_As                       N..L.....LSQ..Q...R.L.......E...    32
MYSS_CHICK      S....S.....AS..H..G.IL.L...LNQIKS...RK.A..D..I.QLK  1598
MYHB_CHICK      ..MKTQ...L.DE.QAA.DAKL.LEVNMQAMKSQFERDL.ARD.QNEEK.  1596

                         760        *       780        *       800
MYS_AEQIR       RNHQRALESMQASLEAEAKGKADAMRIKKKLEQDINELEVALDASNRGKA  1634
MYSA_DROME      K......D..............E.L.M.....A......I...HA.KAN.  1636
MYHTail1_As     K.....ID..........R...E.L.M.....S......I...HA.KAN.   420
MYHTail2_As     K.....ID..............E.L.M.....S......I...H..KAN.    82
MYSS_CHICK      ...L.IV....ST.D..IRSRNE.L.L...M.G.L..M.IQ.SHA..MA.  1648
MYHB_CHICK      .QLLKQ.HEHETE..D.R.QR.L.AAA.....V.VKD..SQV.SA.KARE  1646

                        *       820        *       840        *
MYS_AEQIR       EMEKTVKRYQQQIREMQTSIEEEQRQRDEARESYNMAERRCTLMSGEVEE  1684
MYSA_DROME      .AQ.NI......LKDI..AL.....A..D...QLGIS...ANALQN.L..  1686
MYHTail1_As     .AQ.NI.K..LNLK.I.AAL...T.A...L..Q.A.S..H.NAL...L..   470
MYHTail2_As     .AQ.NI.K..I.LK.T.QAL.....A...V..Q.A.S....NA.H..L..   132
MYSS_CHICK      .AQ.NLRNT.GTLKDT.IHLDDAL.TQEDLK.QVA.V...AN.LQA....  1698
MYHB_CHICK      .AI.QLRKL.A.MKDY.RDLDDARAA.E.IFATAREN.KKAKNLEA.LIQ  1696

                         860        *       880        *       900
MYS_AEQIR       LRAALEQAERARKASDNELADANDRVNELTSQVSSVQGQKRKLEGDINAM  1734
MYSA_DROME      S.TL....D.G.RQAEQ.....HEQL..VSA.NA.ISAA.....SELQTL  1736
MYHTail1_As     S.QL...SD...RTAEA....LHEQ....SAGNA.LSMA.K....EMQ.L   520
MYHTail2_As     S.QL....D...R.A.S...ELHEN....SA.N..LSMA......EMQ.L   182
MYSS_CHICK      ..G....T..S..VAEQ..L..TE..QL.HT.NT.LINT.K...T..VQI  1748
MYHB_CHICK      .QED.AA......QA.L.KEEMAEELASAN.GRT.L.DE..R..AR.AQL  1746

                        *       920        *       940        *
MYS_AEQIR       QTDLDEMHGELKGADERCKKAMADAARLADELRAEQDHSNQVEKVRKNLE  1784
MYSA_DROME      HS....LLN.A.NSE.KA....V...............AQTQ..L..A..  1786
MYHTail1_As     HA.....LN.A.NSE.KA....V..........S..E.AM.Q..M..S..   570
MYHTail2_As     HA.....LN.A.SSE.KA....V.............E.AQ.Q..M..AM.   232
MYSS_CHICK      .SEMEDTIQ.ARN.E.KA...IT...MM.E..KK...T.AHL.RMK..MD  1798
MYHB_CHICK      EEE...E.SNIETMSD.MR..VQQ.EQ.NN..AT.RATAQKN.NA.QQ..  1796
```

```
                              960          *        980          *       1000
MYS_AEQIR       SQVKEFQIRLDEAEASSLKGGKKMIQKLESRVHELEAELDNEQRRHAETQ  1834
MYSA_DROME      Q.I..L.V.......NA.......A.....Q..R...N...G......DA.  1836
MYHTail1_As     Q...DL.V......QAA.......I......K.R...N..E......S.AS   620
MYHTail2_As     G.I..L.V.......AA.......I.....QK.R...T.........S.AA   282
MYSS_CHICK      QT..DLHV......QLA.......QL....A..R...G.V.S..K.S..AV  1848
MYHB_CHICK      R.N..LRSK.Q.M.GAVKSKF.ST.AA..AKIAS..EQ.EQ.A.EKQAAA  1846

                         *      1020          *      1040          *
MYS_AEQIR       KNMRKADRRLKELAFQADEDRKNQERLQELIDKLNAKIKTFKRQVEEAEE  1884
MYSA_DROME      ..L..SE..V...S..SE.....H..M.D.V...QQ....Y...I.....  1886
MYHTail1_As     ..Y...E.....VQ..QE.E...H..M.D.V...QQ....Y...I.....   670
MYHTail2_As     ..V.RSE..V...Q...E........M.D.V...QQ....Y...I.....   332
MYSS_CHICK      .GV..YE..V...TY.CE.....IL...D.V...QM.V.SY...A.....  1898
MYHB_CHICK      .TL.QK.KK..DALL.VEDE..QA.QYKDQAE.G.LRL.QL...L.....  1896

                       1060          *       1080          *       1100
MYS_AEQIR       IAAINLAKYRKAQHELEEAEERADTADSTLQKFRAKSRSSV---------  1925
MYSA_DROME      ...L....F....Q..........L.EQAIS.....G.AG----------  1926
MYHTail1_As     ...L....F..V.Q...D......MSEQ..A.L...N....---------   711
MYHTail2_As     ...L....F..V.Q...D......M.ENVAA.L...N...A---------   373
MYSS_CHICK      LSNV..S.F..I............I.E.QVN.L.V...E-----------  1937
MYHB_CHICK      ESQRIN.NR..L.R..D..T.SN.ALGREVAALKS.L.RGNEPVSFAPPR  1946

                         *      1120          *
MYS_AEQIR       -SVQRSSVSVSASN                          1938
MYSA_DROME      -..G.GASPAPRATSVRPQFDGLAFPPRFDLAPENEF  1962
MYHTail1_As     -.AG.ALSPGP                              721
MYHTail2_As     -..G.AMSPIPMGKPGRPK                      391
MYSS_CHICK      -IHGKKIEEEE                             1947
MYHB_CHICK      R.GG.RVIENATDGGEEEIDGRDGDFNGKASE        1978
```

Supplementary Figure 3 - Alignment of tarantula myosin heavy chain tail fragments with corresponding parts of known myosin heavy chains.

The abbreviation and Swiss-Prot accession number for the myosin heavy chain sequences aligned are: MYS_AEQIR, *Aequipecten irradians* (Bay scallop) myosin heavy chain, striated muscle (P24733); MYSA_DROME, *Drosophila melanogaster* (Fruit fly) myosin heavy chain, muscle (P05661); MYHhead3_S2_As, *Aphonopelma sp.* (Tarantula) skeletal muscle myosin heavy chain head 3 S2 part; MYHTail1_As, *Aphonopelma sp.* (Tarantula) skeletal muscle myosin heavy chain major isoform; MYHTail2_As, *Aphonopelma sp.* (Tarantula) skeletal muscle myosin heavy chain minor isoform; MYSS_CHICK, *Gallus gallus* (Chicken) myosin heavy chain, skeletal muscle, adult (P13538); MYHB_CHICK, *Gallus gallus* (Chicken) myosin heavy chain, gizzard smooth muscle (P10587). Dot indicates identity with the top sequence; Dash indicates a gap inserted for spacing purposes. The skip residues are boxed. The numbering above the sequences (20, 40 etc) starts from the beginning of the coiled-coil tail. The numbering of all MYS are based on true residue numbers except MYHhead3_S2_As.

```
                         *        20         *        40         *        60
ACT1_DROME      MCDEEVAALVVDNGSGMCKAGFAGDDAPRAVFPSIVGRPRHQGVMVGMGQKDSYVGDEAQ   60
ACT2_DROME      ............................................................   60
ACT2_Brazil     ...D........................................................   60
ACT1_Brazil     ...DD.......................................................   60
ACT3_Brazil     ...DD.......................................................   60
ACT2_As                                    .A...............................   33
ACT1_As         ...DD.......................................................   60
ACT3_DROME      ...D........................................................   60
ACT5_DROME      ...D........................................................   60
ACT4_DROME      .....AS.............................................C.......   60
ACT6_DROME      ...DDAG.....................................................   60

                         *        80         *       100         *       120
ACT1_DROME      SKRGILTLKYPIEHGIVTNWDDMEKIWHHTFYNELRVAPEEHPVLLTEAPLNPKANREKM  120
ACT2_DROME      ............................................................  120
ACT2_Brazil     ............................................................  120
ACT1_Brazil     ............................................................  120
ACT3_Brazil     ............................................................  120
ACT2_As         ............................................................   93
ACT1_As         ............................................................  120
ACT3_DROME      ............................................................  120
ACT5_DROME      ............................................................  120
ACT4_DROME      ............................................................  120
ACT6_DROME      ............................................................  120

                         *       140         *       160         *       180
ACT1_DROME      TQIMFETFNTPAMYVAIQAVLSLYASGRTTGIVLDSGDGVSHTVPIYEGYALPHAILRLD  180
ACT2_DROME      ............................................................  180
ACT2_Brazil     ............................................................  180
ACT1_Brazil     ............................................................  180
ACT3_Brazil     ............................................................  180
ACT2_As         ............................................................  153
ACT1_As         .........A..................................................  180
ACT3_DROME      ............................................................  180
ACT5_DROME      .........A..................................................  180
ACT4_DROME      ............................................................  180
ACT6_DROME      ............................................................  180

                         *       200         *       220         *       240
ACT1_DROME      LAGRDLTDYLMKILTERGYSFTTTAEREIVRDIKEKLCYVALDFEQEMATAASSSSLEKS  240
ACT2_DROME      ............................................................  240
ACT2_Brazil     ............................................................  240
ACT1_Brazil     ............................................................  240
ACT3_Brazil     ............................................................  240
ACT2_As         ............................................................  213
ACT1_As         .....................V......................................  240
ACT3_DROME      ....................................................A.......  240
ACT5_DROME      ....................................................A.......  240
ACT4_DROME      ....................................................A.......  240
ACT6_DROME      ....................................................A.......  240
```

```
                      *       260         *       280         *       300
ACT1_DROME   YELPDGQVITIGNERFRCPEALFQPSFLGMEACGIHETTYNSIMKCDVDIRKDLYANTVL  300
ACT2_DROME   ....................S.......................................  300
ACT2_Brazil  ...............................S............................  300
ACT1_Brazil  ...............................S............................  300
ACT3_Brazil  ...............................S............................  300
ACT2_As      ...............................S............................  273
ACT1_As      ....................T..........SV..........Q................  300
ACT3_DROME   ....................S..........S......V..................I..  300
ACT5_DROME   ....................S..........S......V..................I..  300
ACT4_DROME   .................T.............S......V.Q................N..  300
ACT6_DROME   ...............................S......V.....................  300

                      *       320         *       340         *       360
ACT1_DROME   SGGTTMYPGIADRMQKEITALAPSTMKIKIIAPPERKYSVWIGGSILASLSTFQQMWISK  360
ACT2_DROME   ............................................................  360
ACT2_Brazil  ............................................................  360
ACT1_Brazil  ............................................................  360
ACT3_Brazil  ............................................................  360
ACT2_As      ............................................................  333
ACT1_As      ............................................................  360
ACT3_DROME   ...................S........................................  360
ACT5_DROME   ............................................................  360
ACT4_DROME   ............................................................  360
ACT6_DROME   ............................................................  360

                      *
ACT1_DROME   QEYDESGPSIVHRKCF  376
ACT2_DROME   ................  376
ACT2_Brazil  ................  376
ACT1_Brazil  ................  376
ACT3_Brazil  ................  376
ACT2_As      ................  349
ACT1_As      ................  376
ACT3_DROME   ........G.......  376
ACT5_DROME   ........G.......  376
ACT4_DROME   ........G.......  376
ACT6_DROME   ........G.......  376
```

Supplementary Figure 4 - Alignment of tarantula actins with other actins.

The abbreviation and Swiss-Prot accession number for the actin sequences aligned are: ACT1_DROME: *Drosophila melanogaster* (Fruit fly)cytoplasmic actin, Act5C(P10987); ACT2_DROME: *Drosophila melanogaster* (Fruit fly)cytoplasmic actin, Act42A (P02572); ACT3_DROME:*Drosophila melanogaster* (Fruit fly) Actin-57B(P53501); ACT4_DROME: *Drosophila melanogaster* (Fruit fly) Actin, larval muscle(P02574); ACT5_DROME: *Drosophila melanogaster* (Fruit fly) Actin-87E(P10981); ACT6 _DROME: *Drosophila melanogaster* (Fruit fly) Actin, indirect flight muscle(P83967). ACT1_As, ACT2_As, *Aphonopelma sp.* (Tarantula) Actin isoform 1(major) and 2 (minor); ACT1_Brazil, ACT2_Brazil, ACT3_Brazil, the actin isoforms found in hemocytes of *Acanthoscurria gomesiana* (tarantula) based on EST analysis. Dot indicates identity with the top sequence; Dash indicates a gap inserted for spacing purposes.

```
                                        EF site I
                       *        20         *        40         *
TNNC2_DROME   MDNIDEDLTPEQIAVLQKAFNSFDHQKTGSIPTEMVADILRLMGQPFDRQ   50
TNNC3_DROME   .SSV............................................KK   50
TNNC_TACTR     AGAA...SK..VQM.R...DM..RD.K.V.H.N..ST...TL..T.EEK   49
TNNC_lycosa      MV.E.SK..VEM.K...DM..KE.K...N.S..ST...TL..Q.VES   47
TNNC1_As         MV.E.SK..VEM.K...DM..RE.K...H.S..ST...TL..T.VES   47
TNNC1_DROME      MSDE..K..T.L.RN...A..PE.N.Y.N.A..GT..SML.HQL.DA   47
Q175S7_AEDAE      M...DKQ.LEL.RN...A..QE.K.C.G.Q..GT..SML.HQL.DK   46

                          EF site II
                      60         *        80         *       100
TNNC2_DROME   ILDELIDEVDEDKSGRLEFEEFVQLAAKFIVEEDDEAMQKELREAFRLYD  100
TNNC3_DROME   ..E...E............G..............A...............  100
TNNC_TACTR    D.KD..A.I.Q.G..E......MA...R.L....A....E..........   99
TNNC_lycosa   E.K...Q.I.V.G..E...D..LA.T.R.L....S....E.......M..   97
TNNC1_As      E.K...I.I.Q.G..E...D..LA.T.R.L....S....E.......M..   97
TNNC1_DROME   T.ADI.A.....G..QI.....TT...R.L....A...MA..K.......   97
Q175S7_AEDAE  L.K.I.....A.G..E.......T...R.L....A....Q..K.......   96

               EF site III                         EF site IV
                       *       120         *       140         *
TNNC2_DROME   KQGNGYIPTSCLKEILKELDDQLTEQELDIMIEEIDSDGSGTVDFDEFME  150
TNNC3_DROME   .....F...T........................................  150
TNNC_TACTR    ...Q.F.NV.D.RD..RA...K...D...E..A...T.............  149
TNNC_lycosa   .E.....NVRD.R...RA...K...D...E..A...T.............  147
TNNC1_As      .E.....NV.D.R...RA...K...D...E..A...T.............  147
TNNC1_DROME   .E.....T.GV.R...R....K..NDD..M....................  147
Q175S7_AEDAE  .E.....T.QV.R........N..NDD..M....................  146


TNNC2_DROME   MMTGE    155
TNNC3_DROME   .....    155
TNNC_TACTR    ....     153
TNNC_lycosa   ....D    152
TNNC1_As      ....D    152
TNNC1_DROME   V...GDD  154
Q175S7_AEDAE  V...GDD  153
```

Supplementary Figure 5 - Alignment of tarantula TnCs with known TnCs.

The abbreviation and Swiss-Prot or GenBank accession number for the TnC sequences aligned are: TNNC2_DROME, *Drosophila melanogaster* (Fruit fly) Troponin C, isoform 2 (P47948); TNNC3_DROME, *Drosophila melanogaster* (Fruit fly) Troponin C, isoform (P47949); TNNC_TACTR, *Tachypleus tridentatus* (Japanese horseshoe crab) Troponin C (P15159); TNNC_lycosa, *Lycosa singoriensis* (Chinese wolf spider) Troponin C (ABX75382); TNNC1_As, *Aphonopelma sp.* (Tarantula) skeletal muscle Troponin C, isoform 1; TNNC1_DROME, *Drosophila melanogaster* (Fruit fly) Troponin C, isoform 1 (P06742); Q175S7_AEDAE, *Aedes aegypti* (Yellowfever mosquito) Troponin C (Q175S7). Dot indicates identity with the top sequence; Dash indicates a gap inserted for spacing purposes. The EF-hands are labelled.

```
                         *        20         *        40         *
TNNI1_As      MAD-----------------------------------------------    3
TNNI3_As      ...-----------------------------------------------    3
TNNI2_As      ...QS---------------------------------------------    5
TNNI2_DROME   ...D----------------------------------------------    4
TNNI_DROME    ...DEKKAAAPAAAPAAAAKPAAPAAAPAANGKAAPAANGKAAPAAAAAP   50

                      60         *        80         *       100
TNNI1_As      ---------------DAKQKALDEKERKKAEVRARLEAAAAGKKAKKG-F   37
TNNI3_As      ---------------.................................-.   37
TNNI2_As      ---------------EDERRRQE..........R...ETTKF..C...G.   40
TNNI2_DROME   ---------------E..KAKQA.I...R....K.M.E.SKA......-.   38
TNNI_DROME    AGPPKDPNDPKVKAEE..KAKQA.I...R....K.M.E.SKA......-.   99

                         *       120         *       140         *
TNNI1_As      MTPARKKKLRTLLRKKAAEELKREQERKAEERKKTIASRCGPQKNLDGIN   87
TNNI3_As      ..................................................   87
TNNI2_As      ...Q..............................M.TE...QP.S..NA.   90
TNNI2_DROME   ...E......L...........K......A..RRI.EE...SPR..SDAS   88
TNNI_DROME    ...E......L...........K......A..RRI.EE...SPR..SDAS  149

                     160         *       180         *       200
TNNI1_As      EAELINICKEYHDRIAELEGQKYDMEFQARHKEYKINELNIQVSDLRGKF  137
TNNI3_As      ..A.QA......Q..CQ..DA...L.YAV.Q.D.V...............  137
TNNI2_As      ..T.QA.....YK...Q..DD...L.YDV.Q.DFL....T...N......  140
TNNI2_DROME   ....QT...Q.WQ.VYS...D.F.L.HVQKV.AQE..D..A..N......  138
TNNI_DROME    .G..QE..E..YE.MYIC....W.L.YEV.K.DWE..D..A..N......  199

                         *       220         *       240         *
TNNI1_As      VKPVLKKVSRFEYGKFEKLMRAAKKADNDFRTNLKSVGPSTKYKLEDDVK  187
TNNI3_As      ..............................                      167
TNNI2_As      ...T.....KY.-......KMV..TTEV.........-K.N.F..GE.EE  188
TNNI2_DROME   ...A.....KY.-N..A..QK--.A.EFN..NQ..V.-KKKEFT..EEE.  184
TNNI_DROME    ...A.....KY.-N..A..QK--.A.EFN..NQ..V.-KKKEFT..EEE.  245

                     260         *
TNNI1_As      ESK--PEWAAGVKEAAKKGEE       206
TNNI2_As      GK.QA....NK                 199
TNNI2_DROME   .K.--.D.SK.KPGD..VK..VEAEA  208
TNNI_DROME    .K.--.D.SK.KPGD..VK..VEAEA  269
```

Supplementary Figure 6 - Alignment of tarantula TnIs with *Drosophila* TnIs.

The abbreviation and GenBank or Swiss-Prot accession number for the TnIs aligned are: TNNI1_As, TNNI2_As, TNNI3_As, *Aphonopelma sp.* (Tarantula) TnI isoform1, 2 and 3 (partial) ; TNNI2_DROME, *Drosophila melanogaster* (Fruit fly) Troponin I isoform E (NP_728139); TNNI_DROME, *Drosophila melanogaster* (Fruit fly) Troponin I (Tn I) (P36188). Dot indicates identity with the top sequence; Dash indicates a gap inserted for spacing purposes.

```
                     *        20         *        40         *
TNNT_DROME  MSDDEEYTSSEEEEVVEETREETKPPQT--PAEGEGDPEFIKRQDQKRSD   48
TNNT_PERAM  MSDEEEYSEEEEEVPV-----DTKPRHSVIVVEEKGDPEFVKRQEQKSSA   45

                    60         *        80         *       100
TNNT1_As       MWQEYIDQWRKQRAKEEEELRKLKERQARRKVTRAEQEKRLMELKRK   47
TNNT_DROME  LDDQLK...TE.....S...D..K....K..K.......E.QKMAQR.KE   98
TNNT_PERAM  LDEQLK...AE..........D.K...DK.SK...M..DE...MA.R.KQ   95

                     *       120         *       140         *
TNNT1_As    QEEQRVREIEEKKQKEAEAKRKRLEEAERKRQAMLEEQRKQKEGVKPNFV   97
TNNT_DROME  E..R....A.....R.I.E..M......K......QAMKD-.DKKG...T  147
TNNT_PERAM  E..R..........RDI.E..R......K.....MQALKE..QQKG...T  145

                   160         *       180         *       200
TNNT1_As    IQKKAEGGAPVVSHHPGGFDKLSTLEQARNELLKSKEQLAEDKAIALTYR  147
TNNT_DROME  .A..DA.--------------VLG.SS.AM.RN.T....E.E.K.S.SF.  183
TNNT_PERAM  ....DPS---------------FNMSS.QI.RN.T....E.E.K.S.SF.  180

                     *       220         *       240         *
TNNT1_As    VKPLNIEGLGSGKLKDVGEELWNKIVQLESEKYDLEEKMKRQDYDLRELT  197
TNNT2_As                                                   .KS    3
TNNT_DROME  I...A...F.EA..REKAQ...EL..K..T.......RQ.......K..K  233
TNNT_PERAM  I...E..N.NVD...VKAT...DA..K..T.......RQ.......K..K  230

                   260         *       280         *       300
TNNT1_As    ERQKQINRQKALKKGIDPAEAEGKYPPKIHVASKFERRVDRRTFGDKKQF  247
TNNT2_As    K............................S....Y...L.........SL   53
TNNT_DROME  .....QL.H......L..EALT.......Q....Y.....T.SYD...KL  283
TNNT_PERAM  .....QL.H......L..EALT.......Q....Y.....T.SYD...KL  280

                     *       320         *       340         *
TNNT1_As    YDGGLEEDIKAKLEKCWKDRMTSFKERGPKQLPKW----------DPTAP  287
TNNT2_As    FE.................................----------.....   93
TNNT_DROME  FE..WD.IS.DSN..I.NEKKEQYTG.QKSK....FGERPGKKAGE.ET.  333
TNNT_PERAM  FE..WATLSSESN..V..SKYEL.AN.SKSK....FGERPGKKKG..ES.  330

                   360         *       380         *       400
TNNT1_As    KVKEVIEARTYDED--DDLLDLEPPSFGAPAEPEPAPRAPSPPPPQEEEE  335
TNNT2_As    ..............--..................................  141
TNNT_DROME  EGE.DAK.---...IVE.DEEV.EEVVEEED.EAEEDEEEEEEEEE....  380
TNNT_PERAM  E-E.EVK.---.AG-V..--E..E.T.----....E.-E.EEEAAE..A.  368

                     *
TNNT1_As    EEEEEEEEDEEEEEEE   351
TNNT2_As    ................   157
TNNT_DROME  ........E.......E  397
TNNT_PERAM  ........E.......   384
```

Supplementary Figure 7 - Alignment of tarantula TnTs with known arthropoda TnTs.

The abbreviation and Swiss-Prot accession number for the TnTs aligned are: TNNT1_As, TNNT2_As, *Aphonopelma sp.* (Tarantula) TnT isoform1 and 2 (partial); TNNT_DROME, *Drosophila melanogaster* (Fruit fly) Troponin T, skeletal muscle (P19351); TNNT_PERAM, *Periplaneta americana* (American cockroach) Troponin T (Q9XZ71). Dot indicates identity with the top sequence; Dash indicates a gap inserted for spacing purposes.

```
                     *        20         *        40         *
TPM2_As     MEAIKKKMQGMKLEKDNAVDRAETAENQSRDANLRADKAEEEVRSLQKKI   50
TPM2_DROME  .D.......A........I.K.D.C...AK...S....LN....D.E..F   50
TPM_BOOMI   .........A................Q...E.A...E.............   50

                    60         *        80         *       100
TPM2_As     QQIENELDQVQEQLAQANNKLEEKDKALQAAEGEVAALNRRIQLLEEDLE  100
TPM2_DROME  V.V.ID.VTAK...EK..TE....E.L.T.T.S...TQ..KV.QI.....  100
TPM_BOOMI   ..............S...S.............A....H............  100

                     *       120         *       140         *
TPM2_As     RSEERLKTATAKLEEASQAADESERMRKMLEHRSITDEERMDALEDQLKE  150
TPM1_As             ..........................................   42
TPM2_DROME  K....ST..QQ..L..T.S...NN..C.V..N..QQ......Q.TN....  150
TPM_BOOMI   .......I..Q...............................G..G....  150

                   160         *       180         *       200
TPM2_As     ARLMAEEADRKYDEVARKMAMVEADLERAEERAETGENKIVELEEELRVV  200
TPM1_As     ..................................................   92
TPM2_DROME  ..ML..D..T.S...S..L.F..DE..V..D.VRS..S..M......K..  200
TPM_BOOMI   ..T...D...........L..................T............  200

                     *       220         *       240         *
TPM2_As     GNNLKSLEVSEEKALQKEETYEMTIRQMTQRLQEAEARAEFAERSVQKLQ  250
TPM1_As     ..............Q.R..A..GQ..L..S..K.................  142
TPM2_DROME  ..S...........N.RV.EFKREMKTLSIK.K...Q...H..KQ.KR..  250
TPM_BOOMI   .......................Q.....N....................  250

                   260         *       280
TPM2_As     KEVDRLEDELVQEKEKYKAISDELDQTFAELTGY  284
TPM1_As     ..................................  176
TPM2_DROME  ........R.FN........C.D...........  284
TPM_BOOMI   ............................S.....  284
```

Supplementary Figure 8 - Alignment of tarantula TPMs with known arthropoda TPMs.

The abbreviation and Swiss-Prot accession number for the TPMs aligned are: TPM1_As, TPM2_As, *Aphonopelma sp.* (Tarantula) TPM isoform1(major) and 2 (minor); TPM2 _DROME, *Drosophila melanogaster* (Fruit fly) Tropomyosin-2(P09491); TPM_BOOMI, *Boophilus microplus* (Cattle tick) Tropomyosin (O97162). Dot indicates identity with the top sequence; Dash indicates a gap inserted for spacing purposes.
